# Increased Meflin Expression in Cancer-Associated Fibroblasts Restrains Tumor Cell Proliferation and Shapes Vessel-Rich Stroma in Triple-Negative Breast Cancer

**DOI:** 10.1016/j.ajpath.2026.01.006

**Published:** 2026-02-02

**Authors:** Akihiro Sakai, Yuki Miyai, Yukihiro Shiraki, Ryota Ando, Nobutoshi Esaki, Tadashi Iida, Takahiro Sugie, Masahiro Shibata, Toyone Kikumori, Norikazu Masuda, Hiroyoshi Y. Tanaka, Mitsunobu R. Kano, Atsushi Enomoto, Shinji Mii

**Affiliations:** ∗ Department of Molecular Pathology, Nagoya University Graduate School of Medicine, Nagoya, Japan; † Department of Breast and Endocrine Surgery, Nagoya University Graduate School of Medicine, Nagoya; ‡ Department of Breast Surgery, Graduate School of Medicine, Kyoto University, Kyoto; § Department of Pharmaceutical Biomedicine, Graduate School of Medicine, Dentistry, and Pharmaceutical Sciences, Okayama University, Okayama, Japan; ¶ Department of Pharmaceutical Biomedicine, Graduate School of Interdisciplinary Science and Engineering in Health Systems, Okayama University, Okayama; || Department of Molecular Pathology, Graduate School of Biomedical and Health Sciences, Hiroshima University, Hiroshima, Japan

## Abstract

Recent studies have shown that cancer-associated fibroblasts (CAFs), a key component of the tumor microenvironment, are heterogeneous and can be divided into distinct subsets. Although all CAFs were believed to promote tumor progression, recent studies have identified a distinguished subset of tumor-restraining CAFs (rCAFs). It was previously demonstrated that the up-regulation of Meflin (immunoglobulin superfamily containing leucine-rich repeat) expression confers a tumor-restraining role on CAFs in pancreatic, colon, urothelial, and lung cancers. Triple-negative breast cancer (TNBC) is an aggressive type of breast cancer with a poor prognosis. In this study, it was shown that Meflin can be a candidate marker for rCAFs in TNBC. In co-culture experiments with tumor cells and fibroblasts, Meflin overexpression in fibroblasts inhibited tumor cell growth in a three-dimensional culture model and shifted their gene expression profile toward that characteristic of universal or normal fibroblasts. Meflin overexpression in fibroblasts significantly reduced the expression of the chemokine receptor ACKR3 and enhanced that of the prostaglandin synthase PTGDS. This is suggestive of the involvement of these proteins in tumor microenvironment regulation. Furthermore, Meflin deficiency reduced the area of tumor vessels in a TNBC mouse model, highlighting its role in CAF-mediated inhibition of TNBC progression and improvement of drug delivery. Accordingly, Meflin plays a role as a potential functional marker of rCAFs in TNBC.

Triple-negative breast cancer (TNBC), a subtype of breast cancer, lacks the expression of estrogen receptor, progesterone receptor, and human epidermal growth factor receptor 2.^1^^,^^2^ TNBC accounts for approximately 15% of all breast cancer cases. It is characterized by high-grade and rapid progression, earlier age at onset, easy metastasis, and poor prognosis.^3^ TNBC does not respond to endocrine- or molecular-targeted therapies. Basal-type breast cancer, which largely overlaps with TNBC, is associated with *BRCA* mutation. Although TNBC cases with *BRCA* mutations respond to poly (ADP-ribose) polymerase inhibitors, therapeutic targets for other TNBC cases have not been established, and so far, no drug has demonstrated a high level of efficacy.^4^, ^5^, ^6^, ^7^

Cancer-associated fibroblasts (CAFs) are among the most important components of the tumor microenvironment (TME).^8^ Recent advancements in single-cell sequencing have enabled an in-depth classification of CAFs based on their transcriptomes.^9^^,^^10^ In pancreatic cancer, distinct CAF subpopulations, such as myofibroblastic CAFs, inflammatory CAFs, and antigen-presenting CAFs, have been identified. Similarly, CAF subsets have been observed in other cancers, including breast cancer.^11^ These subsets exhibited different roles in the TME, such as immunosuppression, and influenced tumor progression.^12^ The discovery of a novel CAF subset, which seems to suppress rather than promote cancer, further supports the diversity of CAF roles.^8^, ^9^, ^10^, ^11^, ^12^, ^13^ Further evidence has shown the transition of CAF subtypes during cancer progression, in addition to the complexity of the roles of CAFs.

In breast cancer, further subclassifications of CAFs, such as CAF-S1 to CAF-S4, have been made through single-cell analysis.^11^ They show varying abundance in different breast cancer subtypes, such as TNBC. For instance, in TNBC, CAF-S1 is abundant and is involved in immunosuppression.^11^^,^^14^ In a previous study, CAFs were classified as S100A4-positive CAFs and podoplanin-positive CAFs.^15^ Breast cancers with *BRCA* mutations have been reported to exhibit a relatively higher S100A4-positive CAF/podoplanin-positive CAF ratio.^15^ These findings suggest that the nature of CAFs varies according to the breast cancer subtype, and in particular, the CAF composition of TNBC may differ from that of other breast cancers. The poor prognosis of TNBC may be attributed to its characteristic CAF populations; however, the specific roles of CAFs in TNBC remain unclear.

Meflin is a marker of tumor-restraining CAFs in pancreatic cancer.^16^^,^^17^ Meflin, encoded by the immunoglobulin superfamily containing leucine rich repeat (*ISLR*) gene, is a glycosylphosphatidylinositol-anchored membrane protein and has been identified as a specific marker for mesenchymal stem cells and fibroblasts.^18^ Meflin-positive CAFs differentiate into α-smooth muscle actin–positive and Meflin-negative CAFs, which are considered tumor-promoting CAFs.^16^ Meflin is also considered a marker of CAFs in colorectal, lung, urothelial, and kidney cancers.^19^, ^20^, ^21^ In addition, Meflin-positive CAFs promote the efficacy of immune checkpoint blockade therapy.^20^^,^^21^ Meflin has also been implicated in the suppression of fibrotic diseases in various organs, such as the pancreas, heart, and lung.^22^, ^23^, ^24^, ^25^, ^26^ Therefore, Meflin-positive fibroblasts are thought to be essential for maintaining homeostasis in various tissues and organs. However, the role of Meflin-positive CAFs in breast cancer, including TNBC, remains unknown. In addition, the function of the Meflin molecule remains unclear, despite being an area of great interest.

In this study, the focus was on Meflin-positive fibroblasts in mammary glands and breast tumors. Meflin-positive fibroblasts were found not only in adenocarcinomas but also in benign mammary lesions, such as sclerosing adenosis. Importantly, its expression in CAFs tended to increase with malignancy of the lesions. Further *in vitro* and *in vivo* studies showed that the overexpression (OE) of Meflin in fibroblasts induced a phenotypic shift toward a phenotype characteristic of universal fibroblasts, a source of specialized fibroblasts,^27^ and suppression of tumor cell proliferation. However, its deficiency led to a decrease in tumor vessel area in a TNBC mouse model. This finding suggests that Meflin OE in CAFs may exert tumor-suppressive effects. Furthermore, it may be associated with effective drug delivery in TNBC, as previously indicated in other types of malignancies.

## Materials and Methods

### Human Breast Tissue Samples

Surgically resected breast tissue samples from patients with breast tumors were obtained from Nagoya University Hospital (Nagoya, Japan). This study was approved by the ethics committee of Nagoya University Graduate School of Medicine (Nagoya, Japan; approval number: 2017-0127-6).

### *In Situ* Hybridization

RNA *in situ* hybridization (ISH) assays were performed using RNAscope technology (RNAscope 2.5 HD Brown Reagent Kit; Advanced Cell Diagnostics, Newark, CA) on formalin-fixed, paraffin-embedded human and mouse tissue samples. The formalin-fixed, paraffin-embedded sections were deparaffinized and incubated with hydrogen peroxide solution for 10 minutes at room temperature. Slides were boiled in the target retrieval solution for 15 minutes in a steam warmer or boiled for 3 minutes in a pressure cooker (SR-MP300; Panasonic, Kadoma, Japan). Slides were incubated with Protease Plus (Advanced Cell Diagnostics) for 30 minutes at 40°C, with the relevant probes for 2 hours at 40°C in a dry oven (HybEZ II Hybridization System; Advanced Cell Diagnostics), and then successively incubated with Amp-1 to Amp-6 reagents. Staining was visualized with diaminobenzidine, followed by counterstaining with hematoxylin. To detect the expression of multiple mRNAs on the same slides by fluorescent microscopy, RNAscope Multiplex Fluorescent Reagent Kit v2 (Advanced Cell Diagnostics) was used and stained with the fluorophores tyramide signal amplification with cyanine 3 and with cyanine 5 (Akoya Biosciences, Marlborough, MA), followed by counterstaining with DAPI. The samples were observed under a fluorescence microscope (BZ-X710; Keyence, Osaka, Japan). The expression levels were quantified by counting the number of dots in each image. Cells displaying five or more signal dots were determined to be expressed, whereas those with four or fewer signal dots were classified as nonexpressing.^16^^,^^18^ The RNAscope probes (Advanced Cell Diagnostics) used in this study were as follows: human Meflin (ISLR) (NM_005545.3, region 275 to 1322; Catalog No. 455481), human ACTA2 (NM_001613.2, region 10 to 1341; Catalog No. 311811), human PDGFRA (NM_006206.4, region 844 to 1774; Catalog No. 604481), human PDGFRB (NM_002609.3, region 523 to 2984; Catalog No. 548991), human ACKR3 (NM_020311.2, region 184 to 1190; Catalog No. 441451), human PTGDS (NM_000954.5, region 15 to 808; Catalog No. 431471), human DPT (NM_001937.4, region 2 to 934; Catalog No. 443351), human COL15A1 (NM_001855.4; region 326 to 1193; Catalog No. 484001), human PDPN (NM_001006625.1, region 911 to 2054; Catalog No. 539751), and mouse Meflin (Islr) (NM_012043.4, region 763 to 1690; Catalog No. 450041).

### Immunohistochemistry

Human and mouse tissues were fixed in 10% neutral-buffered formalin, dehydrated, and embedded in paraffin. Slides were deparaffinized and boiled in the target retrieval solution (pH 9.0 or 6.0; Leica Biosystems, Wetzlar, Germany) using a pressure cooker (SR-MP300) for 3 minutes to retrieve the antigen. Endogenous peroxidase activity was inhibited by applying 0.3% hydrogen peroxide in methanol for 15 minutes. The slides were blocked with 2.5% normal goat serum for 30 to 60 minutes at room temperature and incubated with primary antibodies for 1 hour at room temperature or overnight at 4°C. Next, the slides were incubated with the secondary antibody using a horseradish peroxidase (HRP) goat anti-mouse (MP-7452; Vector Laboratories, Newark, CA), anti-rabbit (MP-7451; Vector Laboratories), or anti-rat (MP-7404; Vector Laboratories) IgG polymer detection kit for 15 minutes at room temperature. Reaction products were visualized using diaminobenzidine (K3468; Dako/Agilent Technologies, Santa Clara, CA). Subsequently, nuclear counterstaining was performed using hematoxylin.

The primary antibodies used in this study were as follows: anti-HER2/ErbB2 (clone 29D8, 2165, 1:400; Cell Signaling Technology, Danvers, MA), anti-ER (clone 1D5, M7047, 1:400; Dako/Agilent Technologies), rat monoclonal anti-mouse CD31 (clone SZ31, DIA-310, 1:100; Dianova/BIOZOL, Eching, Germany), anti-CD31 (clone JC/70A, M0823, 1:100; Dako/Agilent Technologies), rat monoclonal anti-mouse Ki-67 (clone TEC-3, M7249, 1:100; Dako/Agilent Technologies), anti–α-SMA (clone 1A4, M0852, 1:2000, Dako/Agilent Technologies), and anti-podoplanin (clone D2-40, ab77854, 1:100; Abcam, Cambridge, UK). The anti-Meflin antibody that was developed was diluted 10,000 times with Immuno Shot reagent (Cosmo Bio, Tokyo, Japan) before use. Images were quantified using Fiji ImageJ software version 1.8.0_172/1.53c (*https://imagej.net/software/fiji*).

### Cell Culture

Adult normal human dermal fibroblast (NHDF-Ad) cells were purchased from Lonza (Basel, Switzerland) and cultured in fibroblast growth medium-2 (FGM-2, CC-3132; Lonza) following the manufacturer's instructions. Green fluorescent protein (GFP)–labeled MDA-MB-231 cells (MDA-MB-231-GFP), MDA-MB-231 cells, and HCC1937 cells (all from ATCC, Manassas, VA) were cultured in RPMI 1640 medium (Gibco, Waltham, MA). MDA-MB-468-GFP cells were generated by lentiviral transduction of MDA-MB-468 cells (ATCC) with pLV-puro-CMV-EGFP (VectorBuilder, Chicago, IL) and cultured in Dulbecco's modified Eagle's medium (Nacalai Tesque, Kyoto, Japan). All media were supplemented with 8% fetal bovine serum (Nichirei Biosciences, Tokyo, Japan) except the FGM-2 serum-containing kit. Cells were maintained at 37°C in a humidified incubator with 5% CO_2_.

### RNA Interference

Retroviral vectors expressing shRNAs targeting human Meflin (*ISLR*) were used for gene knockdown as previously described.^18^^,^^23^ Target sequences for human Meflin numbers 1 to 4, human major histocompatibility complex class I, and firefly luciferase have been previously described.^18^ A control sequence provided by Takara Bio USA Inc. (San Jose, CA) and a sequence targeting human major histocompatibility complex class I and firefly luciferase were used as negative controls. The production of retroviral supernatants and infection procedures have also been described previously.^18^ Transduced cells were selected using puromycin (2 μg/mL) for 3 to 5 days to establish stable knockdown lines.

### Vectors and Gene Transfection

Human Meflin (ISLR) cDNA was subcloned into a lentiviral vector, pLV-Puro-CMV > hISLR (*https://www.ncbi.nlm.nih.gov/nuccore/NM_201526.2*; accession number NM_201526.2) (VectorBuilder), as previously described.^23^ The pLV-Puro-CMV > EGFP vector was used as a control vector. To produce lentiviral supernatants, HEK293 cells were seeded in 100-mm cell culture dishes. The cells were then transfected with the pVSV-G and psPAX2 packaging plasmids, along with the pLV-Puro-CMV > hISLR or pLV-Puro-CMV > EGFP vector, using Lipofectamine 2000 reagent (Thermo Fisher Scientific, Waltham, MA). The medium was replaced after 24 hours. The virus-containing supernatants were harvested 48 hours after transfection and used for subsequent infection of the NHDF-Ad cells. Transduced cells were selected using puromycin (2 μg/mL) for 3 to 5 days.

### Scratch Assay

NHDF-Ad cells were seeded in 12-well plates at a density of 8.0 × 10^4^ cells per well. Subsequently, the cells were incubated for 2 hours. MDA-MB-231-GFP cells were seeded on the NHDF-Ad cell layer in each well at 2.0 × 10^4^ cells. After overnight incubation, cells in each well were scratched using a yellow tip. The wells were washed twice with phosphate-buffered saline, and the medium was replaced with Dulbecco's modified Eagle's medium supplemented with 1% fetal bovine serum. Images were captured using a phase-contrast microscope (BZ-X710). The migration of total co-cultured cells was quantified by measuring the percentage of wound area filled using ImageJ software. The migration of MDA-MB-231-GFP cells was quantified by measuring the percentage of GFP-positive areas compared with the initial wound area. The scratch assay was also performed using MDA-MB-468 cells instead of MDA-MB-231 cells.

### Three-Dimensional Invasion Assay

MDA-MB-231-GFP and NHDF-Ad cells were trypsinized. Thereafter, cell suspensions were mixed at a ratio of 1:1 and adjusted to 1000 cells/mL. Subsequently, 30-μL drops of the mixed cell suspension were placed onto the lids of 100-mm dishes and turned over to cover the dish containing 10 mL of phosphate-buffered saline.^28^^,^^29^

Mixed cell spheroids were obtained using hanging drop culture after 7 days. Type I collagen (Nitta Gelatin, Osaka, Japan) was diluted with reconstitution buffer (50 mmol/L of sodium hydroxide, 260 mmol/L of sodium bicarbonate, 200 mmol/L of HEPES) and culture media to a final concentration of 2 mg/mL to prepare a collagen mixture. The spheroids were placed in each well of a 96-well plate and precoated with 30 μL of the collagen mixture per well. After placing the spheroid in the center of the well, 50 μL of the collagen mixture was overlaid onto each well. After incubation at 37°C to solidify the mixture into a gel, 50 μL of the medium was added to each well. Spheroids were cultured at 37°C in 5% CO_2_, and images were taken on days 0, 2, and 4 using a fluorescence microscope (BZ-X710). The invasion size was quantified by measuring the maximum diameter using ImageJ software.

### Three-Dimensional Co-culture Model Using the Cell-Stacking Technique

Three-dimensional (3D) co-culture using the cell-stacking technique was based on previously reported methods.^30^^,^^31^ Briefly, MDA-MB-468 and NHDF-Ad cells were trypsinized and incubated in Tris-buffered saline containing 150 mmol/L of sodium chloride, 0.04 mg/mL of fibronectin (Sigma-Aldrich, Saint Louis, MO), and 0.04 mg/mL of gelatin (Fujifilm Wako Pure Chemical Corp., Osaka, Japan) at room temperature for 30 minutes with gentle rocking. The cells were briefly centrifuged and resuspended in culture media independently. Subsequently, 1.0 × 10^6^ NHDF-Ad cells and 5.0 × 10^3^ TNBC cells (MDA-MB-468, MDA-MB-231, or HCC1937) were mixed and seeded into culture inserts for 24-well plates (353095 and 353047; Corning Inc., Corning, NY) coated with 0.12 mg/mL of fibronectin. After 2 days of culture, 3D tissues were fixed with 4% paraformaldehyde and blocked with Blocking One (Nacalai Tesque Inc., Kyoto, Japan) overnight at 4°C. The 3D tissues were incubated with an anti-multi cytokeratin antibody (clones AE1/AE3, NCL-L-AE1/AE3; Leica Biosystems) diluted 500 times in Blocking One. After washing twice with phosphate-buffered saline, the 3D tissues were incubated with Alexa Fluor–labeled secondary antibodies (Thermo Fisher Scientific) diluted 1:1000 in Blocking One. The 3D tissues were stained with DAPI. After washing twice with phosphate-buffered saline, culture insert membranes were mounted on coverslips using PermaFluor aqueous mounting medium (Lab Vision Corp., Fremont, CA). The samples were observed under a confocal laser microscope (TiE-A1R; Nikon, Tokyo, Japan).

### RNA Sequencing and Data Analysis

Total RNA was extracted from co-cultured cells using an RNeasy Mini Kit (Qiagen, Hilden, Germany), according to the manufacturer's instructions, and was transferred to Takara Bio Inc (Otsu, Japan) for RNA sequencing (RNA-seq). Read alignment to the human reference genome (GRCh38) and gene-level count quantification were conducted by Takara Bio using their standard pipeline. Further analyses were performed using the edgeR package version 4.2.1 (*https://bioconductor.org/packages/release/bioc/html/edgeR.html*) in R version 4.3.2 (*https://www.r-project.org*). Differential gene expression analysis was performed using edgeR. Genes with a false discovery rate <0.05 and an absolute log_2_ fold change >1 were considered significantly differentially expressed.

### Immunofluorescence

Multiplex immunofluorescence staining was performed using the Opal multiplex immunohistochemistry (IHC) kit (NEL810001KT; Akoya Biosciences) according to the manufacturer's instructions. Briefly, formalin-fixed, paraffin-embedded sections were deparaffinized and boiled in a target retrieval solution (pH 9.0; Leica Biosystems) for 30 minutes in an electric kettle. After blocking for 15 minutes, the sections were incubated with primary antibodies for 1 hour at room temperature or 4°C overnight. Next, the slides were incubated with the secondary antibody using the ImmPRESS HRP goat anti-mouse (MP-7452; Vector Laboratories) or anti-rat (MP-7404; Vector Laboratories) IgG polymer detection kit for 15 minutes at room temperature. Signal amplification was achieved using Opal fluorophores (Opal 520, Opal 570, or Opal 690). After each round, antibody stripping was performed by boiling the sections in the target retrieval solution at pH 9.0 using an electric kettle. Nuclei were counterstained with DAPI, and the slides were mounted using PermaFluor aqueous mounting medium. The stained sections were imaged using an inverted fluorescence microscope (BZ-X710).

### ISH Followed by Immunohistochemistry

ISH was performed as described previously, and signals were visualized using diaminobenzidine as the chromogen. After ISH and diaminobenzidine staining, tissue sections were blocked with 2.5% normal horse serum for 30 minutes at room temperature. The sections were then incubated with a primary antibody against E-cadherin (clone 36/E-cadherin, 610182, 1:100; BD Biosciences, Franklin Lakes, NJ) for 1 hour at room temperature. After washing, the slides were incubated with a secondary antibody using the ImmPRESS-AP horse anti-mouse IgG polymer detection kit (MP-5402; Vector Laboratories) for 30 minutes at room temperature. The antibody complexes were visualized using a Vulcan Fast Red Chromogen Kit 2 (BRR805AS; Biocare Medical, Pacheco, CA). Finally, nuclear counterstaining was performed using hematoxylin.

### Animals

All mice were maintained under specific pathogen-free conditions in the Division of Experimental Animals, Nagoya University Graduate School of Medicine. All experimental protocols were approved by the Animal Care and Use Committee of Nagoya University Graduate School of Medicine. Meflin knockout (KO) (*Islr*^*–/–*^) mice were generated as described previously.^18^

*BLG-Cre*;*Brca1*^*fl/fl*^;*Trp53*^*+/−*^ mice were obtained from The Jackson Laboratory (Bar Harbor, ME; strain 012620).^32^ Mice mating started at 8 weeks of age, and mice were allowed to gestate twice to induce breast tumor development. *BLG-Cre*;*Brca1*^*fl/fl*^;*Trp53*^*+/−*^ mice were euthanized when their tumors exceeded a certain size or when they became moribund with severely decreased spontaneous physical activity, an indicator of low survival probability beyond 1 day. Genomic DNA extracted from mouse tails was used for PCR-based genotyping, with primer sequences as described previously.^18^^,^^32^

### Analysis of a Publicly Available Single-Cell RNA-seq Data Set

Publicly available single-cell RNA-seq data from human TNBC (*https://singlecell.broadinstitute.org/single_cell/study/SCP1106/stromal-cell-diversity-associated-with-immune-evasion-in-human-triple-negative-breast-cancer#study-download*; accession number PRJEB35405)^33^ were obtained from the Single Cell Portal (Broad Institute). Data were analyzed in R version 4.1.1 using Seurat version 5.3.0 (*https://cran.r-project.org/web/packages/Seurat/index.html*). The gene-count matrix was imported and processed following the standard Seurat workflow. Because the downloaded data set had already undergone preliminary quality control and filtering by the data providers, no additional filtering thresholds were applied. Data normalization and variance stabilization were performed using SCTransform. Dimensionality reduction was achieved by principal components analysis, followed by uniform manifold approximation and projection. Graph-based clustering was conducted using the FindClusters function with a resolution of 0.5. Cell-type annotation was based on canonical marker genes. Meflin and COL1A1 expression was visualized using FeaturePlot and violin plots on the uniform manifold approximation and projection embedding.

### WST-1 Assay

Cell proliferation was measured using a WST-1 assay (11644807001; Roche, Basel, Switzerland). NHDF-Ad cells were seeded on a 96-well plate at 1.0 × 10^3^ cells per well. The WST-1 assay reagent was added to the cell culture medium and incubated for 2 hours. The absorbance of each well was measured at 440 nm using a microplate reader (PowerScan4; DS Pharma Biomedical, Osaka, Japan).

### Western Blot Analysis

Western blot analysis was performed using a conventional protocol as previously described.^34^ Briefly, cells were lysed with SDS sample buffer (10 mmol/L of Tris-HCl, 2% SDS, 2 mmol/L of EDTA, 0.02% bromophenol blue, 6% glycerol, pH 6.8). Cell lysates were sonicated, boiled for 2 minutes with 2% 2-mercaptoethanol, subjected to SDS-PAGE, and subsequently transferred to polyvinylidene fluoride membranes (Millipore, Bedford, MA). Membranes were blocked and incubated with primary antibody for 1 hour at room temperature. After washing, the membranes were incubated with an HRP-conjugated secondary antibody for 1 hour at room temperature. The membranes were washed, and the bands were visualized using an enhanced chemiluminescence detection kit (GE Healthcare, Buckinghamshire, UK) following the manufacturer's instructions. The primary antibodies used were anti-Meflin (HPA050811, 1:1000; Atlas Antibodies, Swedesboro, NJ) and anti–β-actin (AC-74; Merck, Darmstadt, Germany). The secondary antibodies comprised HRP-conjugated rabbit anti-mouse polyclonal (1:5000; Dako/Agilent Technologies) and HRP-conjugated swine anti-rabbit polyclonal (1:3000; Dako/Agilent Technologies) antibodies.

### Prognostic Analysis Using Publicly Available Data Sets

Prognostic analysis was performed using publicly available transcriptomic data sets deposited in the Gene Expression Omnibus and European Genome-phenome Archive repositories, as previously described.^35^ Kaplan-Meier survival analysis was performed using the online tool Kaplan-Meier Plotter (*https://kmplot.com*, last accessed July 4, 2023). Overall, 4929 breast cancer cases were included. Patients were stratified into Meflin-high and Meflin-low groups based on Meflin (ISLR) mRNA expression levels derived from microarray data using the median expression value as the cutoff. Molecular subtypes of breast cancer were classified according to the PAM50 gene expression signature.^36^

### Statistical Analysis

A two-tailed *t*-test was performed to compare differences between the two groups. One-way analysis of variance was performed to compare differences among three or more groups. Knockdown experiments were analyzed using the Bonferroni post hoc test to compare the differences among the three groups. All statistical analyses and data visualization were performed using R version 4.3.2. Kaplan-Meier survival analysis was conducted using the survival version 3.6-4 (*https://cran.r-project.org/web/packages/survival/index.html*) and survminer version 0.5.0 (*https://cran.r-project.org/web/packages/survminer/index.html*) packages in R. The statistical significance of the survival analysis was calculated using log-rank tests. A *P* ≤ 0.05 was considered statistically significant. Data are expressed as the means ± SD.

## Results

### Meflin Expression Is Associated with a Favorable Prognosis in Patients with TNBC

To investigate the impact of Meflin on the biological behavior of breast cancer subtypes, the association between ISLR mRNA (hereafter referred to as Meflin mRNA) expression and prognosis was first investigated using integrated publicly available data sets deposited in the Gene Expression Omnibus and European Genome-phenome Archive repositories,^35^ as detailed in Materials and Methods. An analysis of all subtypes of breast cancer using the Kaplan-Meier Plotter revealed that the prognosis was significantly better in the Meflin-high group than in the Meflin-low group (Supplemental Figure S1). A similar trend was observed in a cohort of patients with the Basal subtype, which largely overlapped with that of TNBC (Supplemental Figure S1).^35^ In contrast, no such trend was observed in luminal A, luminal B, and human epidermal growth factor receptor 2–enriched subtypes (Supplemental Figure S1). Previous reports have shown that CAFs in TNBC represent a population distinct from those of other subtypes of breast cancer.^11^^,^^15^ Therefore, the present study focused on CAFs of TNBC, with the speculation that Meflin mRNA expression level in CAFs may be associated with the different behavior and drug sensitivity of TNBC compared with other breast cancer subtypes.

### Meflin Expression in Fibroblasts in TNBC and Benign Mammary Lesions

**Figure 1:**
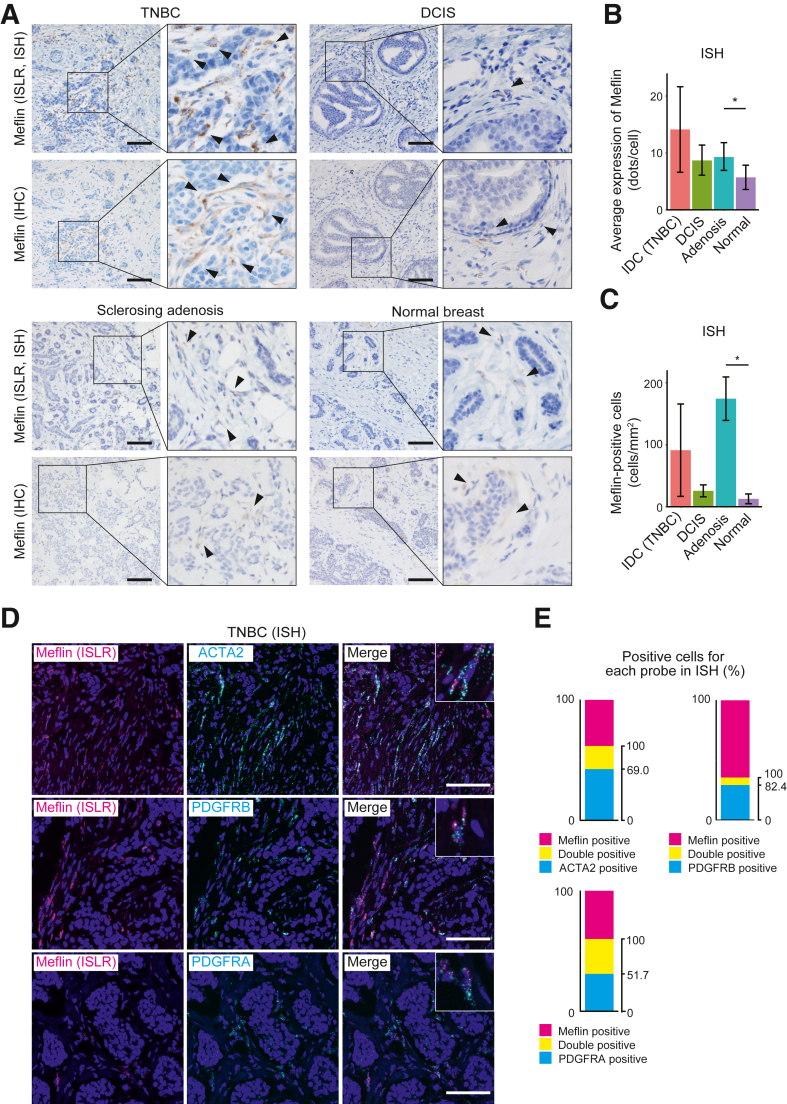
Meflin (immunoglobulin superfamily containing leucine rich repeat [ISLR]) expression in human breast cancer. **A:** Tissue sections from the indicated human breast lesions and normal breast tissue were stained for Meflin mRNA (ISLR) and protein using *in situ* hybridization (ISH) with a Meflin probe (**upper panels**) and immunohistochemistry (IHC) with an anti-Meflin antibody (**lower panels**). Representative images from the same area of the serial sections are shown. The **boxed regions** on the **left panels** are shown at a higher magnification (lower left: ×2.5; others: ×3) in the **right panels**. **Arrowheads** indicate Meflin-positive fibroblasts. **B:** Meflin expression in stromal cells was quantified by calculating the average number of RNA-ISH signal dots per cell. **C:** The number of Meflin-positive cells was quantified by ISH in four types of human breast lesions: invasive ductal carcinoma (IDC), ductal carcinoma *in situ* (DCIS), sclerosing adenosis, and normal tissue. **D:** Representative duplex ISH images of human triple-negative breast cancer (TNBC) tissue. The **upper panels** demonstrate ISH images obtained using probes for Meflin (ISLR) and ACTA2 and their merged images. The **middle** and **lower panels** show ISH images obtained using probes for Meflin (ISLR) and PDGFRB or Meflin and PDGFRA, respectively. The magnified images (×3) are displayed in the **insets**. **E:** The number of cells positive for the indicated markers was counted in (**D**), followed by quantification of the proportion of positive cells. Error bars indicate the standard deviation. *n* = 5 independent samples per group (**B** and **C**). ∗*P* < 0.05. Scale bars = 100 μm (**A** and **D**).

To investigate Meflin expression in human TNBC, a publicly available single-cell RNA-seq data set (*https://singlecell.broadinstitute.org/single_cell/study/SCP1106/stromal-cell-diversity-associated-with-immune-evasion-in-human-triple-negative-breast-cancer#study-download*; accession number PRJEB35405)^33^ was first examined, revealing that Meflin expression is restricted to fibroblasts and not detected in epithelial or immune cell populations (Supplemental Figure S2). Next, Meflin expression was examined in TNBC and ductal carcinoma *in situ* tissues using IHC and ISH. Although many Meflin-positive fibroblasts were detected in TNBC, few Meflin-positive fibroblasts were observed in the stroma surrounding the ductal carcinoma *in situ* lesions (Figure 1A). Meflin-positive fibroblasts were rare in normal breast tissue. Meflin expression was also detected in fibroblasts that proliferated extensively in benign breast lesions, including sclerosing adenosis (Figure 1A). Furthermore, Meflin expression was observed in the neoplastic stromal cells of phyllodes tumors and fibroadenomas (Supplemental Figure S3). Quantification of Meflin expression by IHC analysis showed no significant difference in prognosis between the Meflin-high and Meflin-low groups (Supplemental Figure S4).

Although Meflin expression was detected in fibroblasts across both malignant and benign lesions, the expression levels of Meflin mRNA in individual fibroblasts (measured by the number of ISH dots) tended to be higher in invasive TNBC compared with normal tissues and adenosis lesions (Figure 1B). In contrast, the numbers of Meflin-positive fibroblasts were variable across the normal, benign, and malignant tissues (Figure 1C). Next, the expression of other CAF markers in TNBC was examined using ISH. Meflin mRNA was expressed in CAFs expressing ACTA2, which encodes for α-smooth muscle actin (31.0%), PDGFRB (17.6%), and PDGFRA (48.3%) (Figure 1, D and E). Quantitatively, inverse correlations were observed between Meflin mRNA and the expression of other CAF markers in TNBC CAFs (Supplemental Figure S5). These observations were consistent with findings of previous reports that Meflin-positive fibroblasts first appeared in the early stages of cancer and then differentiated into activated tumor-promoting CAFs, such as α-smooth muscle actin–positive myofibroblasts.^37^^,^^38^

### Meflin Expression in Fibroblasts Has No Apparent Effect on TNBC Tumor Cell Invasion *in Vitro*

ISH data showed that the mRNA expression level of Meflin in individual CAFs tended to be higher in invasive carcinomas than in normal fibroblasts (Figure 1B). This finding implicates the biological significance of elevated Meflin expression in determining CAF phenotype and functionality. Therefore, primary cultured human fibroblasts (NHDF-Ad cells) were transduced with Meflin cDNA to achieve Meflin OE, followed by co-culture with TNBC tumor cell lines (Supplemental Figure S6A). Meflin knockdown NHDF-Ad (shMeflin-1 and shMeflin-2) cells were also generated by shRNA-mediated RNA interference (Supplemental Figure S6B). The proliferation of Meflin OE NHDF-Ad cells was examined using the WST-1 assay. The results showed no significant difference in proliferation between Meflin OE NHDF-Ad cells and control NHDF-Ad cells (Supplemental Figure S6C). No significant differences in cell proliferation were observed among shMeflin-1, shMeflin-2, and shControl NHDF-Ad cells (Supplemental Figure S6D).

**Figure 2:**
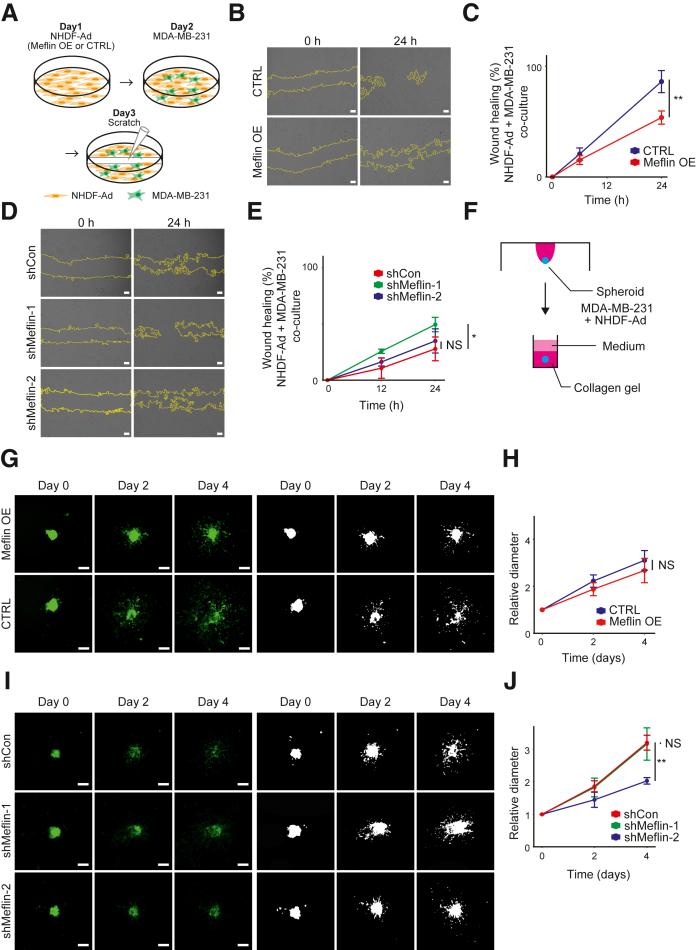
Effects of Meflin overexpression (OE) and depletion in fibroblasts on the migratory response of triple-negative breast cancer cells in two-dimensional and spheroid culture models. **A:** Schematic representation of the scratch assay. Orange cells denote a confluent monolayer of adult normal human dermal fibroblast (NHDF-Ad) cells, and green cells indicate MDA-MB-231 tumor cells seeded on top of the NHDF-Ad cell monolayer. **B:** Co-cultured MDA-MB-231 cells and Meflin OE or control (CTRL) NHDF-Ad cells were subjected to an *in vitro* wound healing assay. Representative images captured 24 hours after wound creation are shown. **Yellow lines** indicate the edges of wound areas. **C:** The percentage of wound area filled 6 and 24 hours after wound creation was quantified using ImageJ software version 1.8.0_172/1.53c. **D:***In vitro* wound healing assay using MDA-MB-231 cells co-cultured with Meflin knockdown (shMeflin-1 or -2) or control (shCon) NHDF-Ad cells. **Yellow lines** indicate the edges of wound areas. **E:** The percentage of wound area filled at 12 and 24 hours after wound creation was quantified using ImageJ software. **F:** Schematic representation of the three-dimensional spheroid invasion assay. **G:** Spheroids were prepared from MDA-MB-231 cells co-cultured with Meflin OE or CTRL NHDF-Ad cells, followed by imaging of spheroids on days 0, 2, and 4 (**left panels**). The corresponding binarized images processed using ImageJ software were used for quantification (**right panels**). **H:** The maximal diameters of MDA-MB-231 spheroids were measured on days 2 and 4, and the relative values are presented as ratios to the average maximal diameter on day 0. NHDF-Ad cells were co-cultured with either Meflin OE or CTRL cells. **I:** Spheroids were prepared from MDA-MB-231 cells co-cultured with Meflin knockdown (shMeflin-1 or -2) or shCon NHDF-Ad cells, and images of the spheroids were captured on days 0, 2, and 4 (**left panels**). Corresponding binarized images processed using ImageJ software were used for quantification (**right panels**). **J:** The maximal diameters of MDA-MB-231 spheroids were measured on days 2 and 4, and the relative values are presented as ratios to the average maximal diameter on day 0. NHDF-Ad cells were co-cultured with shCon, shMeflin-1, or shMeflin-2 cells. *n* = 4 per group (**C** and **E**); *n* = 5 Meflin OE cells (**H**); *n* = 4 CTRL cells (**H**); *n* = 3 shCon cells (**J**); *n* = 4 shMeflin-1 cells (**J**); *n* = 3 shMeflin-2 cells (**J**). Error bars indicate standard deviation. ∗*P* < 0.05, ∗∗*P* < 0.01. Scale bars = 200 μm (**B**, **D**, **G**, and **I**). NS, not significant.

Next, a scratch assay was performed to evaluate the effect of Meflin expression in fibroblasts on the migratory ability of MDA-MB-231, a human TNBC cell line. MDA-MB-231-GFP cells were co-cultured with Meflin OE or shMeflin-1/2 NHDF-Ad cells (Figure 2A). The data showed that Meflin OE NHDF-Ad cells significantly reduced the total migratory ability of the co-cultured cells (Figure 2, B and C). However, there was no significant difference in the migration of GFP-positive MDA-MB-231 cells co-cultured with Meflin OE NHDF-Ad cells compared with that of control NHDF-Ad cells (Supplemental Figure S6, E and F). Meflin knockdown in NHDF-Ad cells exhibited inconsistent effects on the migration of the total cultured cells (Figure 2, D and E, and Supplemental Figure S6, G and H).

A scratch assay was performed using a different TNBC cell line: MDA-MB-468. No significant changes in the migration of MDA-MB-468 cells were observed when co-cultured with Meflin OE or shMeflin-1/2 NHDF-Ad cells compared with control cells (Supplemental Figure S7). A 3D invasion assay was performed by mixing Meflin OE or shMeflin-1/2 NHDF-Ad cells and MDA-MB-231 in a 1:1 ratio, followed by preparation of biomimetic spheroids in collagen using the hanging drop method and measuring the invasive ability of MDA-MB-231 cells (Figure 2F). Similarly, no consistent effects of Meflin OE and shMeflin-1/2 NHDF-Ad cells on the invasive capacity of MDA-MB-231 cells were observed in spheroid culture (Figure 2, G–J). These data indicate that Meflin had no apparent effect on the motility of TNBC tumor cells.

### Meflin Suppresses the Proliferation of TNBC Tumor Cells in a 3D Culture Model that Mimics Fibrotic Tumor Stroma

**Figure 3:**
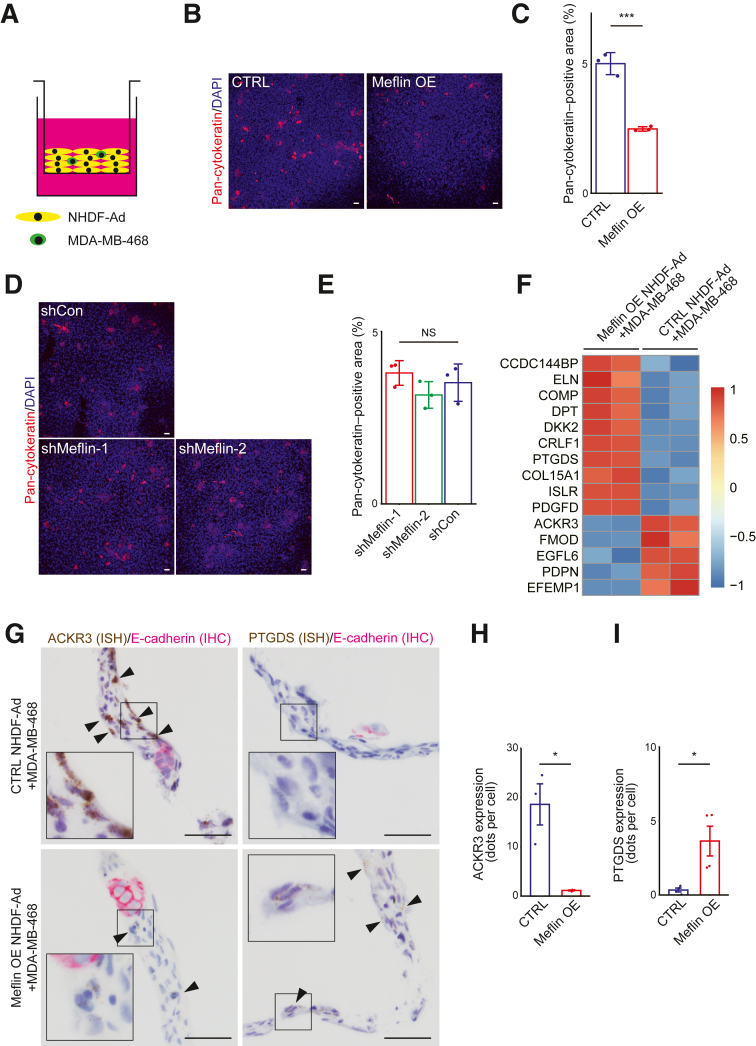
Fibroblasts overexpressing (OE) Meflin suppress triple-negative breast cancer cell proliferation in a biomimetic three-dimensional (3D) culture system. **A:** Schematic representation of the 3D co-culture tumor model using the cell-stacking technique, as described in Materials and Methods. **B:** Representative cross-sectional images of 3D co-culture of MDA-MB-468 cells and Meflin OE or control (CTRL) adult normal human dermal fibroblast (NHDF-Ad) cells. MDA-MB-468 cells were visualized using immunofluorescence staining for pan-cytokeratin (red). Nuclei were visualized using DAPI staining. **C:** Quantification of pan-cytokeratin–positive areas using ImageJ software version 1.8.0_172/1.53c in **B**. **D:** Representative cross-sectional images of 3D co-culture comprising MDA-MB-468 cells and Meflin knockdown (shMeflin-1 or -2) or CTRL (shControl [shCon]) NHDF-Ad cells. **E:** Quantification of pan-cytokeratin–positive areas using ImageJ software in **D**. **F:** MDA-MB-468 cells were co-cultured with either Meflin OE or CTRL NHDF-Ad cells in the 3D co-culture tumor model, followed by the isolation of total RNA and RNA sequencing (RNA-seq) analysis. The heatmap displays the 15 genes with the highest expression variability across samples. **G:** Formalin-fixed, paraffin-embedded samples were prepared from 3D co-culture tumor models, followed by *in situ* hybridization (ISH) for ACKR3 or PTGDS (brown) and immunohistochemistry (IHC) for E-cadherin (red). **Arrowheads** denote NHDF-Ad cells positive for either ACKR3 or PTGDS. **Boxed regions** are shown at higher magnification (×2.5) in **insets** within the same images. **H** and **I:** The number of ISH signal dots for either ACKR3 (**H**) or PTGDS (**I**) per cell was counted and quantified. *n* = 3 per group (**C** and **E**); *n* = 2 per group (**F**); *n* = 3 mean samples per group (**H**); *n* = 4 mean samples per group (**I**). Error bars indicate standard deviation. ∗*P* < 0.05, ∗∗∗*P* < 0.001. Scale bars = 50 μm (**B**, **D**, and **G**). NS, not significant.

Subsequently, another 3D co-culture model was adopted using a cell-stacking technique. This allows for proper recapitulation of the fibrotic stroma found in aggressive human cancers, including TNBC.^30^^,^^31^ In this model, TNBC tumor cells (MDA-MB-468, 5.0 × 10^3^ cells) and NHDF-Ad cells (1.0 × 10^6^ cells) were mixed and seeded into a cell culture insert, allowing the cells to stack and form a 3D structure (Figure 3A). Pan-cytokeratin (AE1/AE3) staining of MDA-MB-468 cells was imaged by confocal microscopy to capture z-stacks containing the entire cells, revealing that Meflin OE NHDF-Ad cells significantly suppressed tumor cell proliferation (Figure 3, B and C). In contrast, shMeflin-1/2 NHDF-Ad cells showed no significant effect on tumor cell proliferation (Figure 3, D and E). Moreover, 3D co-culture experiments using other TNBC cell lines (MDA-MB-231 and HCC1937) consistently demonstrated that Meflin OE NHDF-Ad cells significantly suppressed tumor cell proliferation; conversely, shMeflin-1/2 NHDF-Ad cells exhibited no significant effect (Supplemental Figure S8).

To investigate the molecular changes associated with the tumor-suppressive effects of Meflin OE NHDF-Ad cells, RNA-seq was performed using total RNA isolated from MDA-MB-468 and NHDF-Ad cells in 3D co-culture. The data have been deposited in the Gene Expression Omnibus under accession number GSE301570 (*https://www.ncbi.nlm.nih.gov/geo/query/acc.cgi?acc=GSE301570*). Notably, PDPN, a well-known marker of tumor-promoting CAFs, was down-regulated, whereas DPT and COL15A1, which are markers of universal fibroblasts that are almost equivalent to normal fibroblasts and a source of different types of fibroblasts,^39^ were up-regulated by Meflin OE in NHDF-Ad cells (Figure 3F). These observations were further corroborated by the result of the ISH analysis of cross sections prepared from MDA-MB-468 and NHDF-Ad cells in the 3D co-culture (Supplemental Figure S9). These findings suggest that high-level expression of Meflin is crucial for skewing CAFs toward a more universal fibroblast-like state.

An additional noteworthy finding from the RNA-seq analysis was that Meflin OE altered the expression of several genes involved in the regulation of TME (Figure 3F). For example, the mRNA expression levels of the atypical chemokine receptor 3 (*ACKR3*) and prostaglandin D2 synthase (*PTGDS*) genes were significantly down-regulated and up-regulated, respectively, in Meflin OE NHDF-Ad cells (Figure 3F). ACKR3, also known as C-X-C chemokine receptor type 7, is a receptor for the chemokine C-X-C motif chemokine ligand 12 and has been associated with tumor-promoting activity,^40^^,^^41^ whereas PTGDS plays a tumor-suppressive role.^42^, ^43^, ^44^, ^45^ This observation was further corroborated using ISH, confirming the result of RNA-seq (Figure 3, G–I). The expression of ACKR3 and PTGDS in CAFs in human TNBC samples was also confirmed by ISH (Supplemental Figure S10). These changes in ACKR3 and PTGDS expression were also observed using RNA-seq analysis of Meflin OE NHDF-Ad cells cultured alone, without MDA-MB-468 cells (Supplemental Figure S11). This suggests that Meflin may cell-autonomously regulate ACKR3 and PTGDS expression in fibroblasts to suppress tumor growth or modulate TME.

### Meflin Deficiency and Tumor Vessel Area Reduction in a TNBC Mouse Model

**Figure 4:**
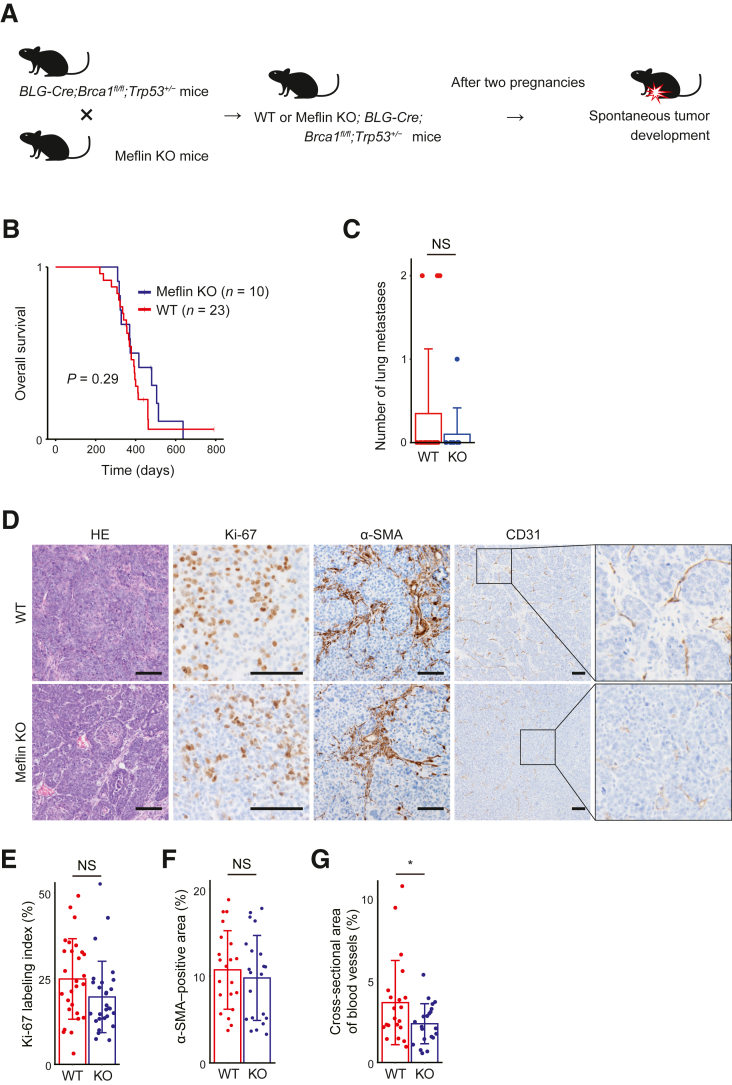
Meflin deficiency reduced the tumor blood vessel area in a triple-negative breast cancer (TNBC) mouse model. **A:** Schematic representation of the experimental design using an autochthonous TNBC mouse model. Meflin knockout (KO) mice were crossed with *BLG-Cre*;*Brca1*^fl/fl^;*Trp53*^+/−^ mice, which spontaneously developed TNBC. **B:** Survival rates of Meflin KO and wild-type (WT) *BLG-Cre*;*Brca1*^fl/fl^;*Trp53*^+/−^ mice were analyzed using the Kaplan-Meier method. **C:** Number of lung metastases in Meflin KO and WT *BLG-Cre*;*Brca1*^fl/fl^;*Trp53*^+/−^ mice. **D:** Representative histologic images of breast tumors derived from WT (**upper panels**) and Meflin KO (**lower panels**) *BLG-Cre*;*Brca1*^fl/fl^;*Trp53*^+/−^ mice. Hematoxylin and eosin staining and representative images of immunohistochemical staining of Ki-67, α-SMA, and CD31 are shown. **Boxed regions** are shown at higher magnification (×4) in the panels on the **right**. **E** and **F:** Percentages of Ki-67–positive tumor cells (**E**) and α-SMA–positive areas (**F**) were quantified using ImageJ software version 1.8.0_172/1.53c. **G:** Areas surrounded by CD31-positive endothelial cells were measured and quantified using ImageJ software. Breast tumors from WT and Meflin KO *BLG-Cre*;*Brca1*^fl/fl^;*Trp53*^+/−^ mice were analyzed in (**E–G**). *n* = 10 Meflin KO *BLG-Cre*;*Brca1*^fl/fl^;*Trp53*^+/−^ mice (**B** and **C**); *n* = 23 WT *BLG-Cre*;*Brca1*^fl/fl^;*Trp53*^+/−^ mice (**B** and **C**); *n* = 6 WT *BLG-Cre*;*Brca1*^fl/fl^;*Trp53*^+/−^ mice (**E–G**); *n* = 6 Meflin KO *BLG-Cre*;*Brca1*^fl/fl^;*Trp53*^+/−^ mice (**E–G**). Error bars indicate standard deviation. ∗*P* < 0.05. Scale bars = 100 μm (**D**). NS, not significant.

Given the finding that Meflin OE in fibroblasts suppressed the proliferation of TNBC tumor cells in the 3D culture model, the *in vivo* role of Meflin was analyzed. This was performed using an autochthonous TNBC mouse model and Meflin KO mice. For the TNBC model, *BLG-Cre*;*Brca1*^fl/fl^;*Trp53*^+/−^ mice, which spontaneously develop TNBC, were used.^32^ *BLG-Cre*;*Brca1*^fl/fl^;*Trp53*^+/−^ mice were mated with wild-type and Meflin KO mice, and the overall survival of the mice and the histology of the developed breast tumors were analyzed (Figure 4A). The tumors were confirmed to be TNBC using IHC (data not shown). No apparent differences were observed in tumor growth time, tumor tissue morphology, or overall survival between Meflin KO and wild-type mice (Figure 4B and Supplemental Figure S12). No significant difference was observed in the number of lung metastases between the groups (Figure 4C).

Given the possible involvement of Meflin in chemokine and prostaglandin signaling pathways that are relevant in shaping the TME (Figure 3, F–I), tumor cell proliferation, amount of stroma, and blood vessels in the developed tumors were analyzed by IHC. Although there were no significant differences in tumor cell proliferation (Ki-67–labeling index) or the number of CAFs labeled by α-smooth muscle actin, which marks a large subset of CAFs, the cross-sectional area of blood vessels in the tumors, as analyzed by IHC using anti-CD31 antibody, was significantly reduced in tumors developed in Meflin KO mice compared with those in wild-type mice (Figure 4, D–G).

**Figure 5:**
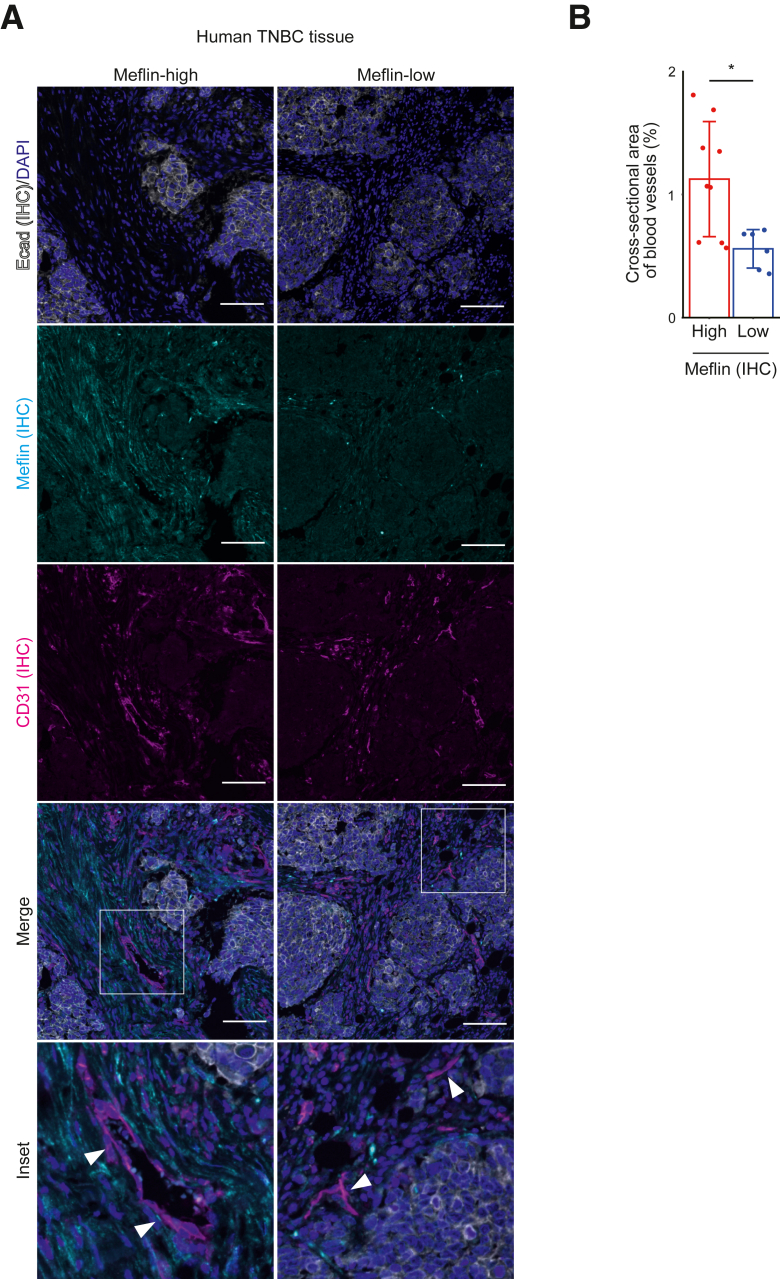
Correlation between Meflin expression in cancer-associated fibroblasts and tumor blood vessel areas in human triple-negative breast cancer (TNBC). **A:** Tissue sections from the Meflin-high (**left panels**) and Meflin-low (**right panels**) regions of human TNBC cases were stained using the indicated antibodies by multiplex immunofluorescence. **Arrowheads** indicate CD31-positive endothelial cells. **Boxed regions** are shown at higher magnification (×2.5) in the **bottom panels**. **B:** Quantification of the percentage of the area surrounded by CD31-positive endothelial cells using ImageJ software version 1.8.0_172/1.53c. Three TNBC cases were analyzed, including Meflin-high lesions and Meflin-low lesions. *n* = 9 Meflin-high lesions (**B**); *n* = 6 Meflin-low lesions (**B**). Error bars indicate standard deviation. ∗*P* < 0.05. Scale bars = 100 μm (**A**). IHC, immunohistochemistry.

To confirm the results from the TNBC mouse model, the areas of CD31-positive tumor vessels were quantified in tissue sections obtained from the Meflin-high and Meflin-low regions of human TNBC cases. Consistent with the results observed in mice, the tumor blood vessel areas were significantly larger in the Meflin-high regions than in the Meflin-low regions of human TNBC (Figure 5).

## Discussion

In this study, it was shown that the phosphatidylinositol-anchored membrane protein Meflin is expressed in CAFs, a key component of TME in TNBC, and may suppress tumor cell proliferation in a 3D context. Although Meflin OE showed no effect on tumor cell growth in two-dimensional culture, it significantly decreased proliferation in a 3D culture model that closely mimics the fibrotic stroma of aggressive human cancers, such as TNBC. This finding suggests a tumor-inhibitory role of Meflin expressed in CAFs and aligns with the correlation between high Meflin expression and favorable breast cancer prognosis (Supplemental Figure S1). An important finding was that an increased expression of Meflin, but not its basal expression, in fibroblasts is essential for them to exert tumor-restraining functions (Figure 3, A–E).

Although a genetically engineered autochthonous TNBC mouse model did not reveal any apparent effect of Meflin expression in CAFs on tumor progression, Meflin deficiency induced a decrease in tumor vessel area. This finding is consistent with previous studies that showed that pharmacologic induction of Meflin expression in CAFs by the synthetic retinoid Am80 increased the tumor vessel area and improved drug delivery in mouse models of pancreatic and urothelial cancers.^17^^,^^21^ Although the mechanism by which Meflin increases tumor vessel area remains unclear, previous reports indicated that Meflin enhances bone morphogenetic protein 7 signaling and inhibits lysyl oxidase, an enzyme critical for collagen cross-linking and fibrosis, thereby suppressing fibrogenesis.^17^^,^^19^ The resulting reduction in tissue stiffness and interstitial pressure could mechanically enlarge the tumor vessel lumen.

RNA-seq analysis demonstrated that Meflin OE induced a phenotypic shift in fibroblasts from a CAF-like state toward a profile more closely resembling that of universal fibroblasts.^39^ This result supports the notion that increasing Meflin expression could provide a strategy for reprogramming CAFs to exhibit a more tumor-suppressive or homeostatic state. Notably, Meflin OE also altered the expression levels of *ACKR3* and *PTGDS,* genes known to regulate tumor angiogenesis and growth. ACKR3, a receptor for C-X-C motif chemokine ligand 12, has been linked to tumor-promoting signaling and angiogenesis.^40^^,^^41^ Prostaglandin D_2_, synthesized by the enzyme encoded by the *PTGDS* gene, suppresses angiogenesis by reducing endothelial permeability and acts as a tumor suppressor.^42^, ^43^, ^44^, ^45^, ^46^, ^47^ In the present mouse model, Meflin expression was associated with increased tumor vessel area, suggesting that its role in vascular formation and angiogenesis may differ from that of prostaglandin D_2_. Another hypothesis is that Meflin attenuates fibrosis and, together with prostaglandin D_2_–mediated reductions in vascular permeability, contributes to vascular normalization, which improves drug delivery.^48^^,^^49^

Meflin expression was also found in fibroblasts that proliferate in benign breast lesions, such as sclerosing adenosis, suggesting its role in the inflammatory stromal reaction in the development of both neoplastic and nonneoplastic breast diseases. Considering the data in the present study and previous studies that Meflin has a tumor-suppressive role in various types of cancer, it is tempting to speculate that Meflin-positive fibroblasts have a reparative role in benign fibroinflammatory diseases. This issue will be addressed by further analyses using mouse models of benign breast lesions. Interestingly, Meflin expression in CAFs varied between ductal carcinoma *in situ* and invasive TNBC, indicating its involvement in the evolution of breast cancer pathology. The inverse correlations between Meflin and other CAF markers, such as ACTA2 and PDGFRB, suggest a potential role of Meflin in the differentiation of fibroblasts into activated CAFs, a critical process in tumor progression (Supplemental Figure S4).

A limitation of this study is that it provides only observational data on how Meflin expression varies across different stages and subtypes of breast cancer. Further comprehensive analyses are necessary to gain a deeper understanding of the role of Meflin in the progression and treatment response of breast cancer. The present *in vitro* experiments showed that Meflin OE, but not KO or knockdown, led to significant phenotypic changes in fibroblasts and affected tumor cell proliferation. Therefore, the induction of Meflin expression in CAFs is significant in controlling cancer progression. Hence, future studies should focus on creating mouse models of Meflin OE to clarify its mechanistic role and therapeutic potential in the TME.

In conclusion, this study highlights the multifaceted role of Meflin in breast cancer, particularly in TNBC. The influence of Meflin expression in CAFs on tumor prognosis, CAF characteristics, cell proliferation, and tumor vessel formation underscores its potential as a therapeutic target.

## Disclosure Statement

None declared.

## Supplemental Data

Supplemental Figure S1 Survival analysis of patients with breast cancer stratified based on Meflin expression levels. Publicly available data were used to analyze the overall survival of patients with breast cancer (All subtypes) or the indicated subtypes of breast cancer by Kaplan-Meier Plotter after stratification into Meflin-high and Meflin-low groups based on Meflin mRNA expression levels, as detailed in Materials and Methods. Basal, basal-like subtype; HER2, human epidermal growth factor receptor 2–enriched subtype; HR, hazard ratio; LumA, luminal A subtype; LumB, luminal B subtype; normal-like, normal breast-like subtype.

Supplemental Figure S2 Meflin expression analysis of a publicly available single-cell RNA sequencing data set of human breast cancer. **A:** Uniform manifold approximation and projection (UMAP) visualization of single-cell RNA sequencing data from human triple-negative breast cancer (TNBC) tissues (*https://singlecell.broadinstitute.org/single_cell/study/SCP1106/stromal-cell-diversity-associated-with-immune-evasion-in-human-triple-negative-breast-cancer#study-download*; accession number PRJEB35405), with clusters colored according to cell type. **B:** Expression pattern of Meflin mRNA (**left panel**) and COL1A1 (**right panel**) in the UMAP plots. **C:** Violin plot showing Meflin mRNA expression across cell types. ISLR, immunoglobulin superfamily containing leucine rich repeat.

Supplemental Figure S3 Meflin expression in fibroepithelial and phyllodes tumors. Serial tissue sections obtained from the indicated fibroadenomas and phyllodes tumors were stained for Meflin mRNA (immunoglobulin superfamily containing leucine rich repeat [ISLR]) by *in situ* hybridization (ISH; **upper panels**) and for Meflin protein by immunohistochemistry (IHC; **lower panels**). Representative images are shown. The **boxed regions** in the **left panels** are shown at a higher magnification (upper right: ×4.5; lower right: ×6; others: ×6.5) in the **right panels**. Scale bars = 100 μm.

Supplemental Figure S4 Detection of Meflin expression in human breast cancer by immunohistochemistry and its association with clinical outcomes. **A:** The proportion score (PS) was assigned based on the percentage of Meflin-positive fibroblasts in the tumor stroma: 0, 0% fibroblasts; 1, 1% to 10% fibroblasts; 2, 11% to 33% fibroblasts; 3, 34% to 66% fibroblasts; and 4, 67% to 100% fibroblasts. The intensity of Meflin staining in fibroblasts was scored as 0 to 2+ (0, negative; 1+, weak; and 2+, strong). Representative images of each intensity score (IS) are shown in the **middle panels**. The total score (TS) was calculated by summing the PS and IS scores. Cases with TSs >3 were considered Meflin-high. **B:** Overall survival (**left panel**) and relapse-free survival (**right panel**) of patients with high (*n* = 44) or low (*n* = 110) triple-negative breast cancer were analyzed using the Kaplan-Meier method. **C:** Overall survival (**left panel**) and relapse-free survival (**right panel**) of patients with Meflin-high (*n* = 37) or Meflin-low (*n* = 119) breast cancer, including all subtypes, in a different cohort (*n* = 156) were analyzed using the Kaplan-Meier method. Meflin expression was determined as described in **A**. Scale bars = 100 μm (**A**).

Supplemental Figure S5 Inverse correlations of expression between Meflin and other cancer-associated fibroblast (CAF) markers in human triple-negative breast cancer (TNBC). **A–C:** Tissue sections from human TNBC were stained for the indicated probes for CAF markers by *in situ* hybridization, followed by quantification of the correlations of expression between Meflin mRNA (immunoglobulin superfamily containing leucine rich repeat [ISLR]) and ACTA2 (**A**), PDGFRB (**B**), and PDGFRA (**C**). The expression levels were quantified by counting the signal dots in the CAFs (Figure 1D). Linear regression analysis was performed to analyze the correlation between Meflin mRNA (ISLR) and ACTA2, PDGFRA, and PDGFRB expression. Note that fibroblasts with >30 dots were classified as ">30." The **red lines** represent best-fit linear regression lines.

Supplemental Figure S6 Effects of Meflin overexpression (OE) and knockdown in adult normal human dermal fibroblast (NHDF-Ad) cells on the migration of MDA-MB-231 cells in a two-dimensional co-culture model. **A:** NHDF-Ad cells were transfected with Meflin OE or control (CTRL) vectors. Representative images of immunoblot analysis for Meflin are shown. **B:** NHDF-Ad cells were transfected with Meflin shRNA (shMeflin-1, -2, -3, or -4) or CTRL shRNA (shCon, shLuc, or shMHC). Representative images of immunoblot analysis for Meflin showed that shMeflin-1 and -2 effectively knocked down Meflin expression in NHDF-Ad cells. **C:** Proliferation of Meflin OE or CTRL NHDF-Ad cells was measured by WST-1 assay. **D:** Proliferation of Meflin knockdown or CTRL NHDF-Ad cells was measured by WST-1 assay. **E–H:** Green fluorescent protein (GFP)–labeled MDA-MB-231 (MDA-MB-231-GFP) cells were co-cultured with Meflin OE or CTRL NHDF-Ad cells (**E** and **F**), or Meflin knockdown or CTRL NHDF-Ad cells (**G** and **H**), followed by scratching of the cell monolayers and measurement of GFP-positive areas to assess the migration of MDA-MB-231-GFP cells. The percentage of GFP-positive area compared with the initial wound area is shown in the graphs (**F** and **H**). Error bars indicate the standard deviation. *n* = 4 per group (**C** and **D**). ∗∗*P* < 0.01. Scale bars = 200 μm (**E** and **G**). NS, not significant.

Supplemental Figure S7 Effects of Meflin overexpression (OE) and knockdown in adult normal human dermal fibroblast (NHDF-Ad) cells on the migration of MDA-MB-468 cells in a two-dimensional co-culture model. **A:***In vitro* wound healing assays using MDA-MB-468 cells co-cultured with Meflin OE or control (CTRL) NHDF-Ad cells. Representative images captured 24 hours after wound creation are shown. **Yellow lines** indicate the edges of wound areas. **B:** Migration of green fluorescent protein (GFP)–labeled MDA-MB-468 cells was observed in the co-culture with Meflin OE or CTRL NHDF-Ad cells. **White lines** indicate the edges of the wound areas. **C:** The migration of total co-cultured cells was quantified by measuring the percentage of wound areas filled 6 and 24 hours after wound creation using ImageJ software version 1.8.0_172/1.53c in **A** (**upper graph**). The migration of GFP-labeled MDA-MB-468 cells was quantified by measuring the percentage of GFP-positive areas compared with the initial wound area 6 and 24 hours after wound creation in **B** (**lower graph**). **D:***In vitro* wound healing assays using MDA-MB-468 cells co-cultured with Meflin knockdown (shMeflin-1 or -2) or CTRL (shControl [shCon]) NHDF-Ad cells. **E:** Migration of GFP-labeled MDA-MB-468 cells was observed in co-culture with Meflin knockdown or CTRL NHDF-Ad cells. **F:** The migration of total co-cultured cells was quantified by measuring the percentage of wound area filled 12 and 24 hours after wound creation using ImageJ software in **D** (**upper graph**). The migration of GFP-labeled MDA-MB-468 cells was quantified by measuring the percentage of GFP-positive area compared with the initial wound area 12 and 24 hours after wound creation in **E** (**lower graph**). Error bars indicate standard deviation. *n* = 4 per group (**C** and **F**). ∗∗*P* < 0.01. Scale bars = 200 μm (**A**, **B**, **D**, and **E**). NS, not significant.

Supplemental Figure S8 Effects of Meflin overexpression (OE) and knockdown in fibroblasts on the proliferation of MDA-MB-231 and HCC1937 cells in a biomimetic three-dimensional (3D) culture system. **A:** Representative cross-sectional images of 3D co-culture of MDA-MB-231 cells and Meflin OE or control (CTRL) adult normal human dermal fibroblast (NHDF-Ad) cells. MDA-MB-231 cells were visualized using immunofluorescence staining for pan-cytokeratin (red). Nuclei were visualized using DAPI staining. **B:** Quantification of pan-cytokeratin–positive areas using ImageJ software version 1.8.0_172/1.53c in **A**. **C:** Representative cross-sectional images of 3D co-culture of MDA-MB-231 cells and Meflin knockdown (shMeflin-1 or -2) or CTRL (shControl [shCon]) NHDF-Ad cells. **D:** Quantification of pan-cytokeratin–positive areas using ImageJ software in **C**. **E:** Representative cross-sectional images of 3D co-culture of HCC1937 cells and Meflin OE or CTRL NHDF-Ad cells. **F:** Quantification of pan-cytokeratin–positive areas using ImageJ software in **E**. **G:** Representative cross-sectional images of 3D co-culture of HCC1937 cells and Meflin knockdown (shMeflin-1 or -2) or CTRL (shCon) NHDF-Ad cells. **H:** Quantification of pan-cytokeratin–positive areas using ImageJ software in **G**. Error bars indicate standard deviation. *n* = 3 per group (**B**, **D**, **F**, **H**). ∗*P* < 0.05, ∗∗*P* < 0.01. Scale bars = 50 μm (**A**, **C**, **E**, and **G**). NS, not significant.

Supplemental Figure S9 Meflin overexpression (OE) induces changes in gene expressions of COL15A1, DPT, and podoplanin (PDPN) in fibroblasts co-cultured with MDA-MB-468 cells in a biomimetic three-dimensional (3D) culture system. **A:** Formalin-fixed, paraffin-embedded samples were prepared from 3D co-culture tumor models, followed by *in situ* hybridization (ISH) for COL15A1, DPT, or PDPN (brown) and immunohistochemistry (IHC) for E-cadherin (red). **Arrowheads** denote adult normal human dermal fibroblast (NHDF-Ad) cells positive for DPT, COL15A1, or PDPN. **Boxed regions** are shown at higher magnification (×2.5) in **insets** within the same images. **B–D:** The number of ISH signal dots for COL15A1 (**B**), DPT (**C**), or PDPN (**D**) per cell was counted and quantified. Data represent mean values of *n* = 4 samples per group. Error bars indicate standard deviation. ∗*P* < 0.05, ∗∗∗*P* < 0.001. Scale bars = 50 μm (**A**). ISLR, immunoglobulin superfamily containing leucine rich repeat.

Supplemental Figure S10 ACKR3 and PTGDS are expressed in the stromal cells of human triple-negative breast cancer (TNBC). Tissue sections from human TNBC were stained for ACKR3 (**upper panels**) or PTGDS (**lower panels**) by *in situ* hybridization (ISH). Representative ISH images are presented. The **boxed regions** in the **left panels** are shown at higher magnification (×2.5) in the **right panels**. The **arrowheads** indicate ACKR3- or PTGDS-positive fibroblasts. Scale bars = 100 μm.

Supplemental Figure S11 RNA sequencing data from adult normal human dermal fibroblast (NHDF-Ad) cells cultured alone in a three-dimensional (3D) culture model using a cell-stacking technique. RNA sequencing was performed on total RNA samples isolated from Meflin overexpressing (OE) and control (CTRL) NHDF-Ad cells that were subjected to the 3D culture model. The heatmap displays 17 genes, consisting of the 15 highest variably expressed genes across samples and CCDC144BP and PTGDS, both of which were markedly altered also in co-culture with MDA-MB-468 cells. *n* = 2 per group.

Supplemental Figure S12 Meflin deficiency did not affect overall survival of the triple-negative breast cancer mouse model. Survival rates of Meflin knockout (KO) and wild-type (WT) *BLG-Cre*;*Brca1*^fl/fl^;*Trp53*^+/−^ mice were analyzed using the Kaplan-Meier method based on days after tumor formation. *n* = 10 KO mice; *n* = 23 WT mice.
