# Supplementary figures and images for: Increased Meflin Expression in Cancer-Associated Fibroblasts Restrains Tumor Cell Proliferation and Shapes Vessel-Rich Stroma in Triple-Negative Breast Cancer
Source: Am J Pathol. 2026 Feb 2;196(5):1205–19. doi: 10.1016/j.ajpath.2026.01.006 (PMC13197952; doi:10.1016/j.ajpath.2026.01.006)

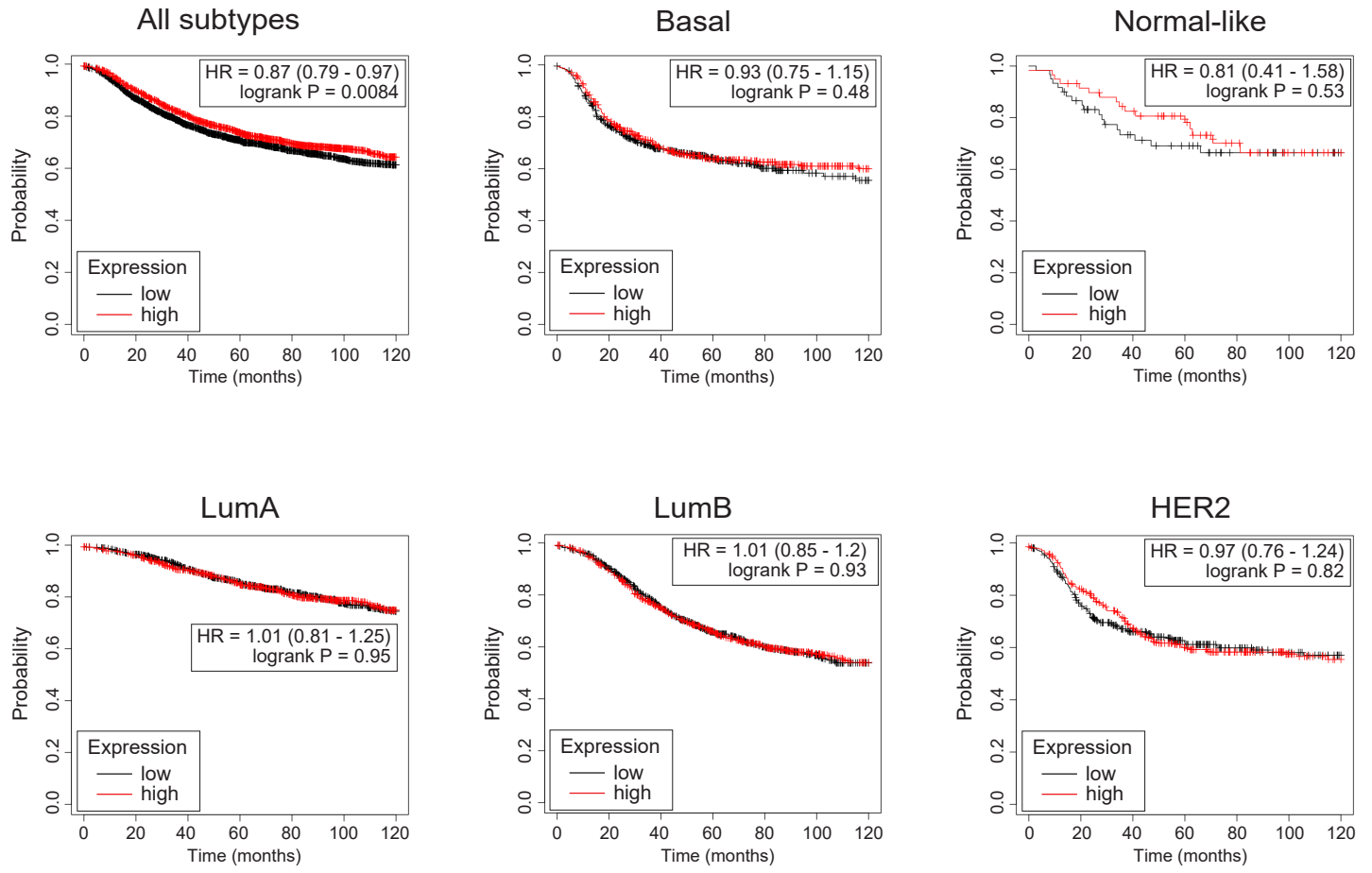

Supplement: Supplemental Figure S1 — Survival analysis of patients with breast cancer stratified based on Meflin expression levels. Publicly available data were used to analyze the overall survival of patients with breast cancer (All subtypes) or the indicated subtypes of breast cancer by Kaplan-Meier Plotter after stratification into Meflin-high and Meflin-low groups based on Meflin mRNA expression levels, as detailed in Materials and Methods. Basal, basal-like subtype; HER2, human epidermal growth factor receptor 2–enriched subtype; HR, hazard ratio; LumA, luminal A subtype; LumB, luminal B subtype; normal-like, normal breast-like subtype. [file mmc1.pdf]

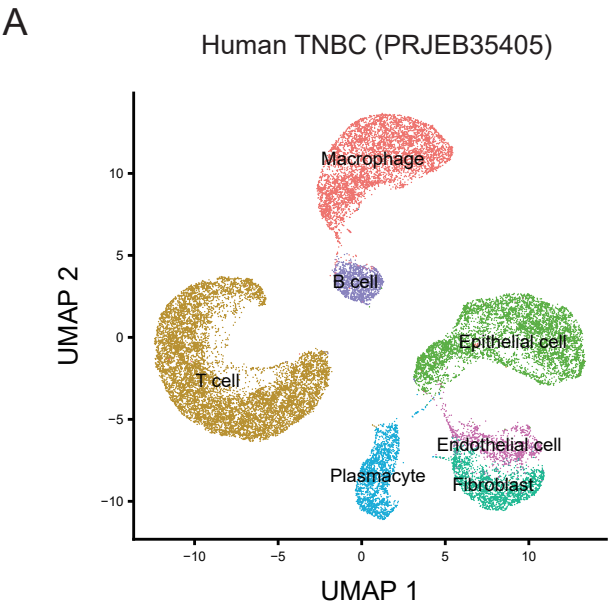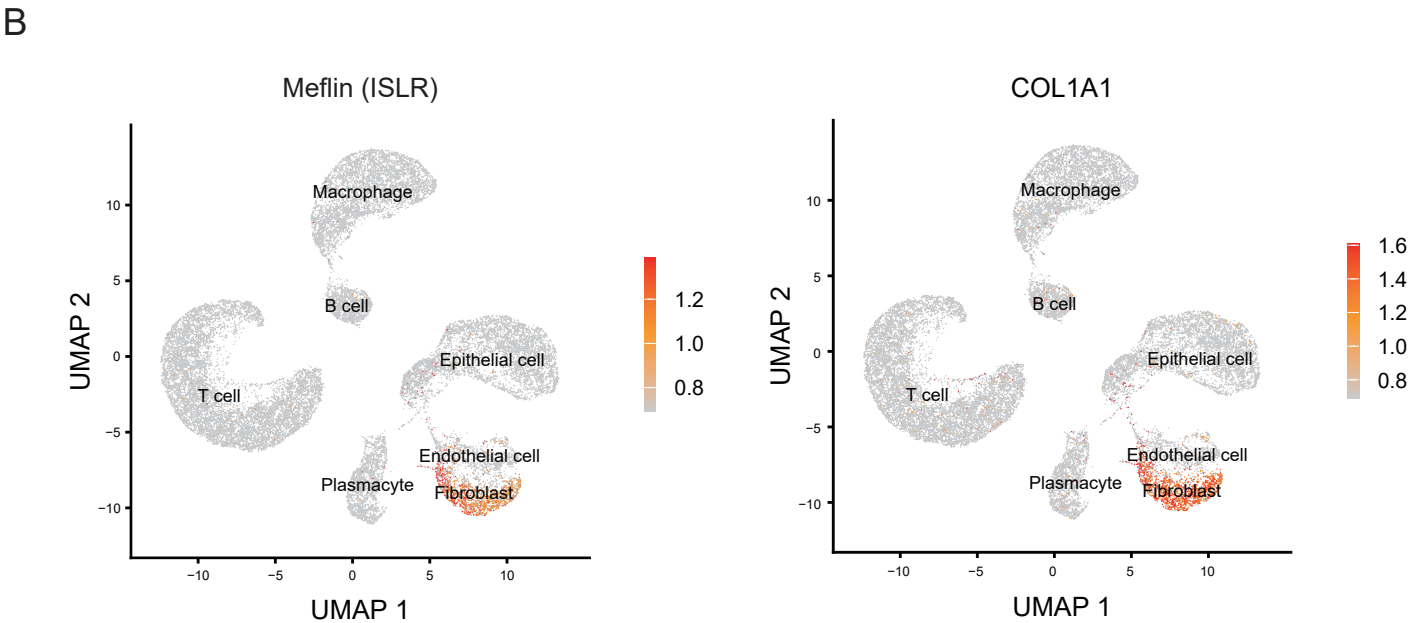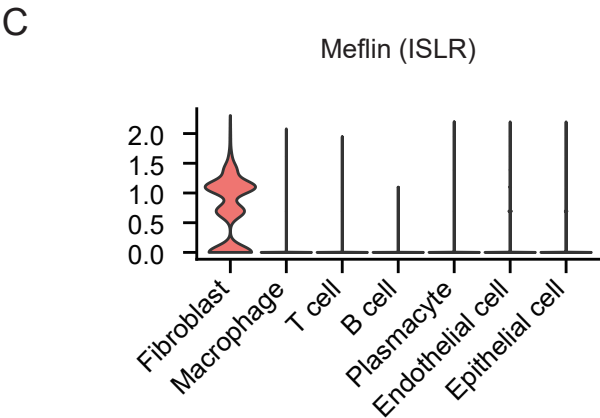

Supplement: Supplemental Figure S2 — Meflin expression analysis of a publicly available single-cell RNA sequencing data set of human breast cancer. A: Uniform manifold approximation and projection (UMAP) visualization of single-cell RNA sequencing data from human triple-negative breast cancer (TNBC) tissues (https://singlecell.broadinstitute.org/single_cell/study/SCP1106/stromal-cell-diversity-associated-with-immune-evasion-in-human-triple-negative-breast-cancer#study-download; accession number PRJEB35405), with clusters colored according to cell type. B: Expression pattern of Meflin mRNA (left panel) and COL1A1 (right panel) in the UMAP plots. C: Violin plot showing Meflin mRNA expression across cell types. ISLR, immunoglobulin superfamily containing leucine rich repeat. [file mmc2.pdf]

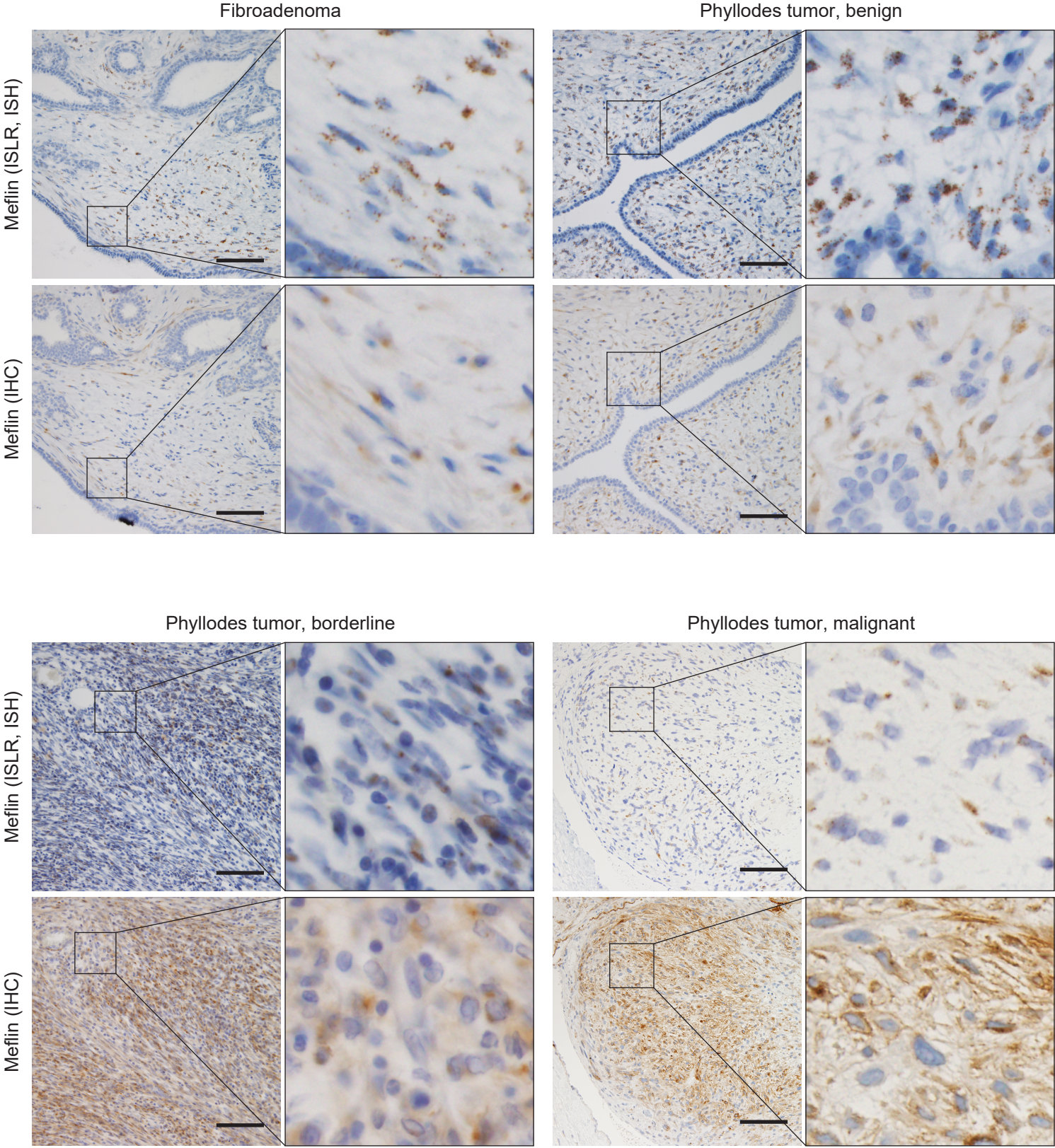

Supplement: Supplemental Figure S3 — Meflin expression in fibroepithelial and phyllodes tumors. Serial tissue sections obtained from the indicated fibroadenomas and phyllodes tumors were stained for Meflin mRNA (immunoglobulin superfamily containing leucine rich repeat [ISLR]) by in situ hybridization (ISH; upper panels) and for Meflin protein by immunohistochemistry (IHC; lower panels). Representative images are shown. The boxed regions in the left panels are shown at a higher magnification (upper right: ×4.5; lower right: ×6; others: ×6.5) in the right panels. Scale bars = 100 μm. [file mmc3.pdf]

A

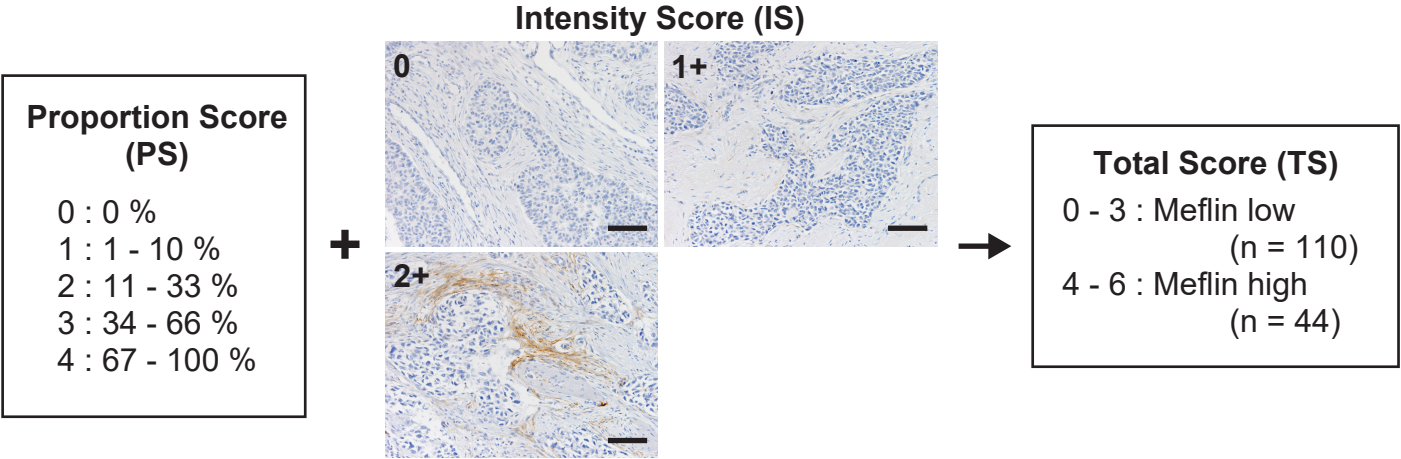

B

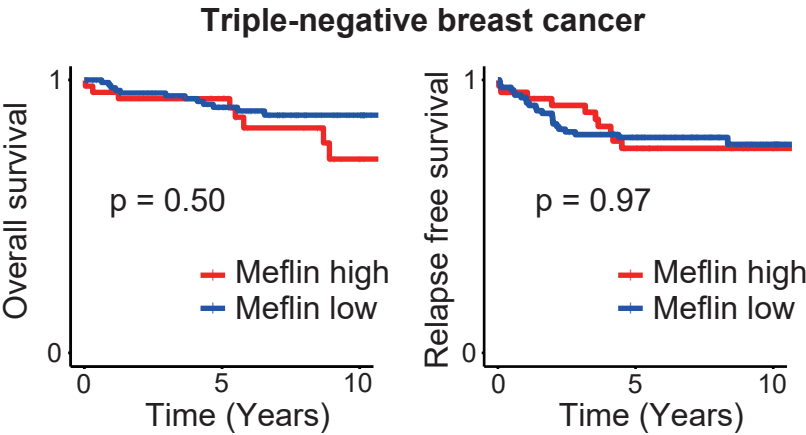

C

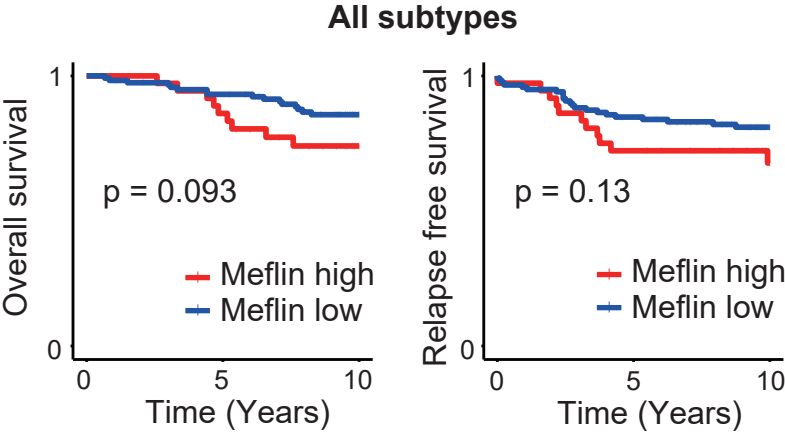

Supplement: Supplemental Figure S4 — Detection of Meflin expression in human breast cancer by immunohistochemistry and its association with clinical outcomes. A: The proportion score (PS) was assigned based on the percentage of Meflin-positive fibroblasts in the tumor stroma: 0, 0% fibroblasts; 1, 1% to 10% fibroblasts; 2, 11% to 33% fibroblasts; 3, 34% to 66% fibroblasts; and 4, 67% to 100% fibroblasts. The intensity of Meflin staining in fibroblasts was scored as 0 to 2+ (0, negative; 1+, weak; and 2+, strong). Representative images of each intensity score (IS) are shown in the middle panels. The total score (TS) was calculated by summing the PS and IS scores. Cases with TSs >3 were considered Meflin-high. B: Overall survival (left panel) and relapse-free survival (right panel) of patients with high (n = 44) or low (n = 110) triple-negative breast cancer were analyzed using the Kaplan-Meier method. C: Overall survival (left panel) and relapse-free survival (right panel) of patients with Meflin-high (n = 37) or Meflin-low (n = 119) breast cancer, including all subtypes, in a different cohort (n = 156) were analyzed using the Kaplan-Meier method. Meflin expression was determined as described in A. Scale bars = 100 μm (A). [file mmc4.pdf]

A

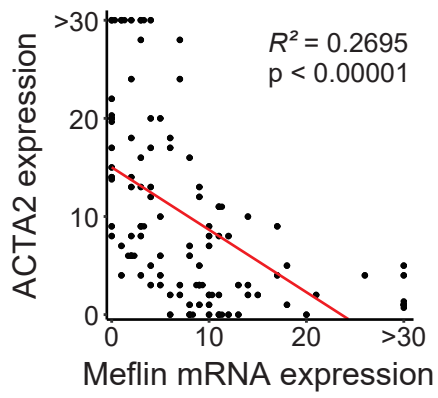

B

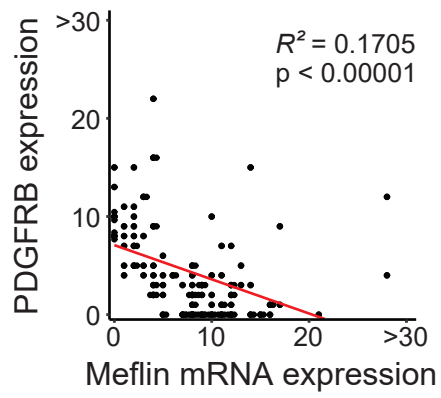

C

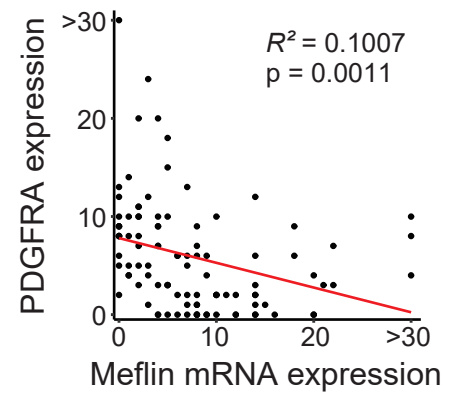

Supplement: Supplemental Figure S5 — Inverse correlations of expression between Meflin and other cancer-associated fibroblast (CAF) markers in human triple-negative breast cancer (TNBC). A–C: Tissue sections from human TNBC were stained for the indicated probes for CAF markers by in situ hybridization, followed by quantification of the correlations of expression between Meflin mRNA (immunoglobulin superfamily containing leucine rich repeat [ISLR]) and ACTA2 (A), PDGFRB (B), and PDGFRA (C). The expression levels were quantified by counting the signal dots in the CAFs (Figure 1D). Linear regression analysis was performed to analyze the correlation between Meflin mRNA (ISLR) and ACTA2, PDGFRA, and PDGFRB expression. Note that fibroblasts with >30 dots were classified as ">30." The red lines represent best-fit linear regression lines. [file mmc5.pdf]

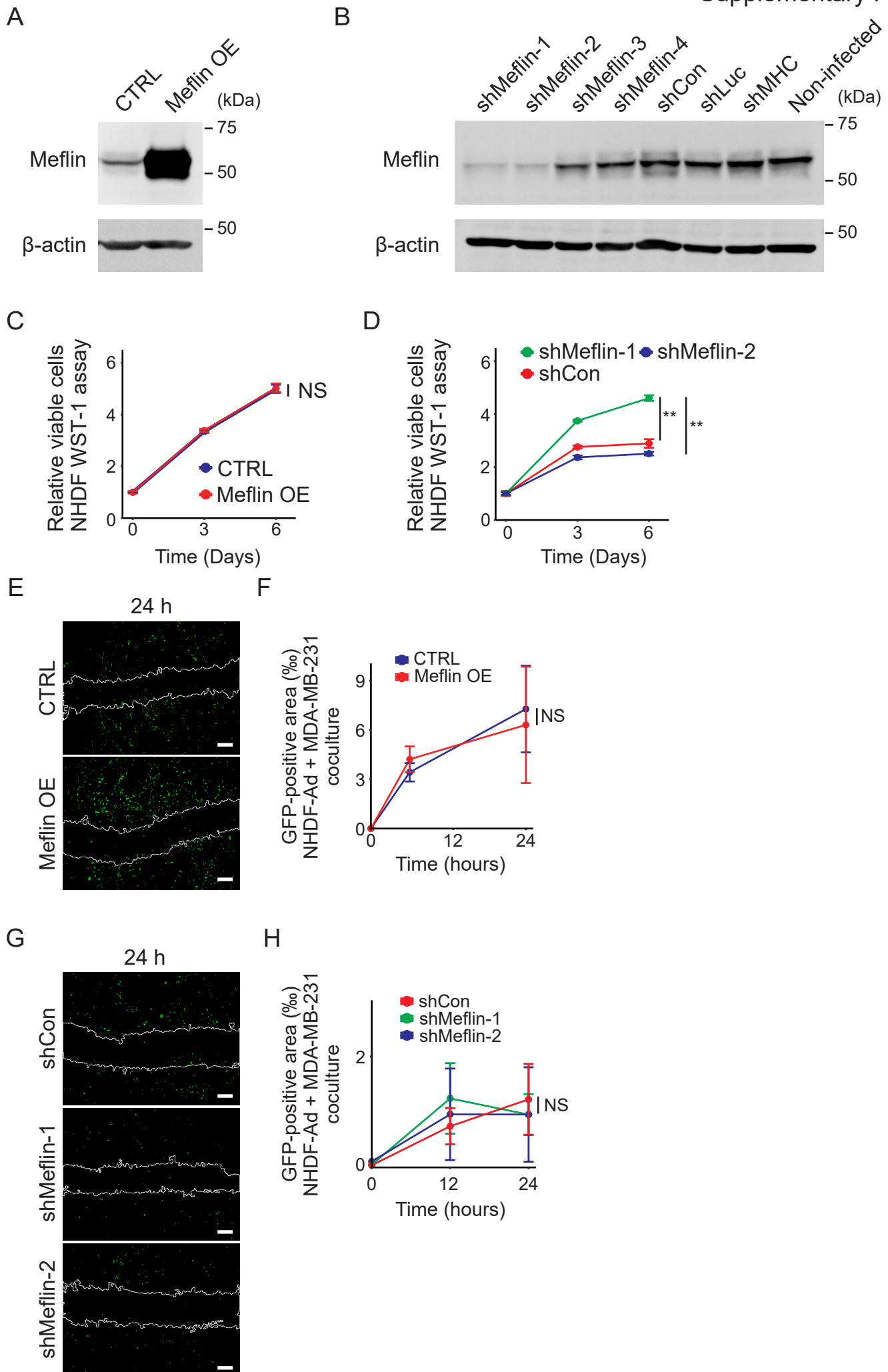

Supplement: Supplemental Figure S6 — Effects of Meflin overexpression (OE) and knockdown in adult normal human dermal fibroblast (NHDF-Ad) cells on the migration of MDA-MB-231 cells in a two-dimensional co-culture model. A: NHDF-Ad cells were transfected with Meflin OE or control (CTRL) vectors. Representative images of immunoblot analysis for Meflin are shown. B: NHDF-Ad cells were transfected with Meflin shRNA (shMeflin-1, -2, -3, or -4) or CTRL shRNA (shCon, shLuc, or shMHC). Representative images of immunoblot analysis for Meflin showed that shMeflin-1 and -2 effectively knocked down Meflin expression in NHDF-Ad cells. C: Proliferation of Meflin OE or CTRL NHDF-Ad cells was measured by WST-1 assay. D: Proliferation of Meflin knockdown or CTRL NHDF-Ad cells was measured by WST-1 assay. E–H: Green fluorescent protein (GFP)–labeled MDA-MB-231 (MDA-MB-231-GFP) cells were co-cultured with Meflin OE or CTRL NHDF-Ad cells (E and F), or Meflin knockdown or CTRL NHDF-Ad cells (G and H), followed by scratching of the cell monolayers and measurement of GFP-positive areas to assess the migration of MDA-MB-231-GFP cells. The percentage of GFP-positive area compared with the initial wound area is shown in the graphs (F and H). Error bars indicate the standard deviation. n = 4 per group (C and D). ∗∗P < 0.01. Scale bars = 200 μm (E and G). NS, not significant. [file mmc6.pdf]

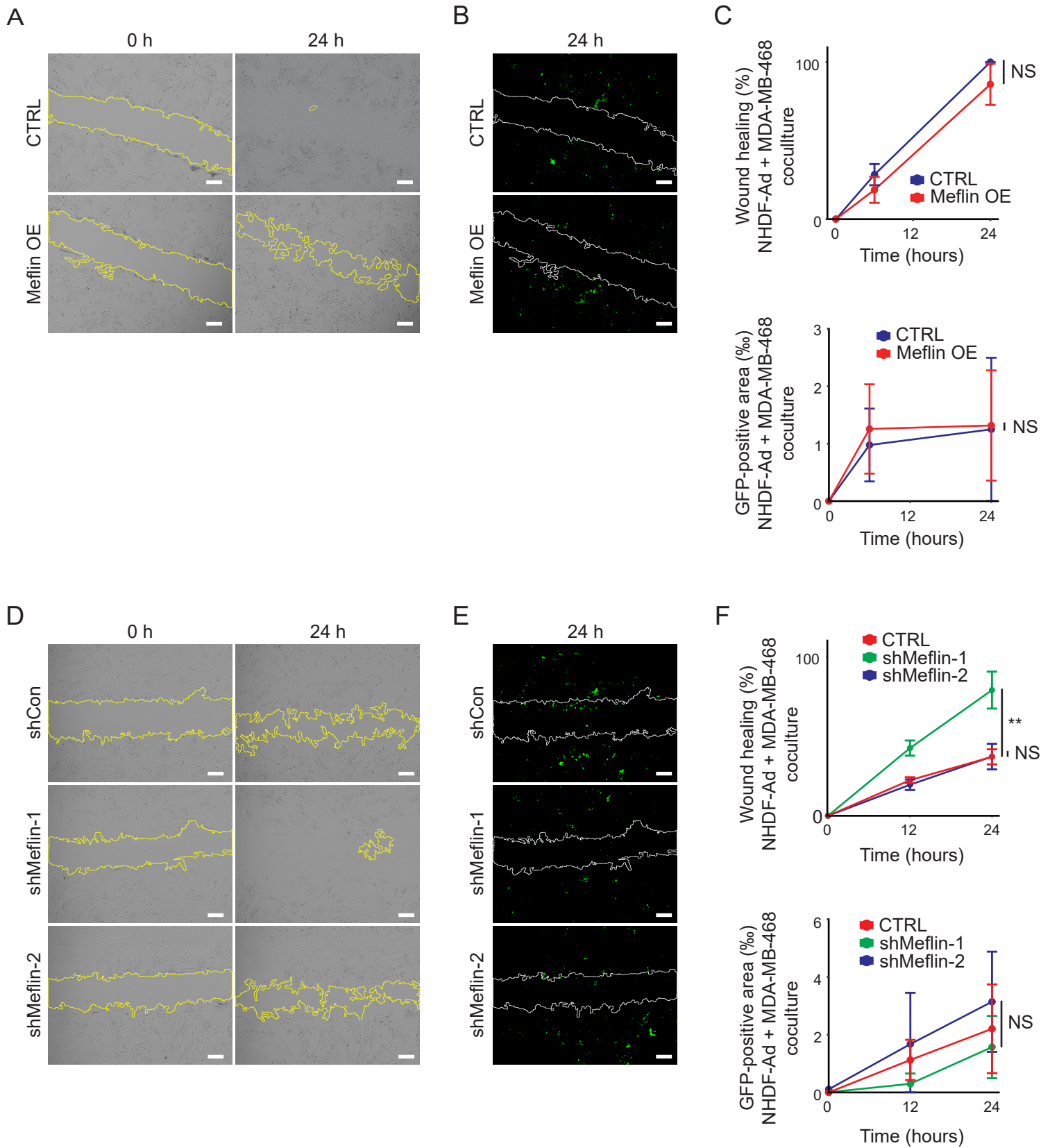

Supplement: Supplemental Figure S7 — Effects of Meflin overexpression (OE) and knockdown in adult normal human dermal fibroblast (NHDF-Ad) cells on the migration of MDA-MB-468 cells in a two-dimensional co-culture model. A:In vitro wound healing assays using MDA-MB-468 cells co-cultured with Meflin OE or control (CTRL) NHDF-Ad cells. Representative images captured 24 hours after wound creation are shown. Yellow lines indicate the edges of wound areas. B: Migration of green fluorescent protein (GFP)–labeled MDA-MB-468 cells was observed in the co-culture with Meflin OE or CTRL NHDF-Ad cells. White lines indicate the edges of the wound areas. C: The migration of total co-cultured cells was quantified by measuring the percentage of wound areas filled 6 and 24 hours after wound creation using ImageJ software version 1.8.0_172/1.53c in A (upper graph). The migration of GFP-labeled MDA-MB-468 cells was quantified by measuring the percentage of GFP-positive areas compared with the initial wound area 6 and 24 hours after wound creation in B (lower graph). D:In vitro wound healing assays using MDA-MB-468 cells co-cultured with Meflin knockdown (shMeflin-1 or -2) or CTRL (shControl [shCon]) NHDF-Ad cells. E: Migration of GFP-labeled MDA-MB-468 cells was observed in co-culture with Meflin knockdown or CTRL NHDF-Ad cells. F: The migration of total co-cultured cells was quantified by measuring the percentage of wound area filled 12 and 24 hours after wound creation using ImageJ software in D (upper graph). The migration of GFP-labeled MDA-MB-468 cells was quantified by measuring the percentage of GFP-positive area compared with the initial wound area 12 and 24 hours after wound creation in E (lower graph). Error bars indicate standard deviation. n = 4 per group (C and F). ∗∗P < 0.01. Scale bars = 200 μm (A, B, D, and E). NS, not significant. [file mmc7.pdf]

A

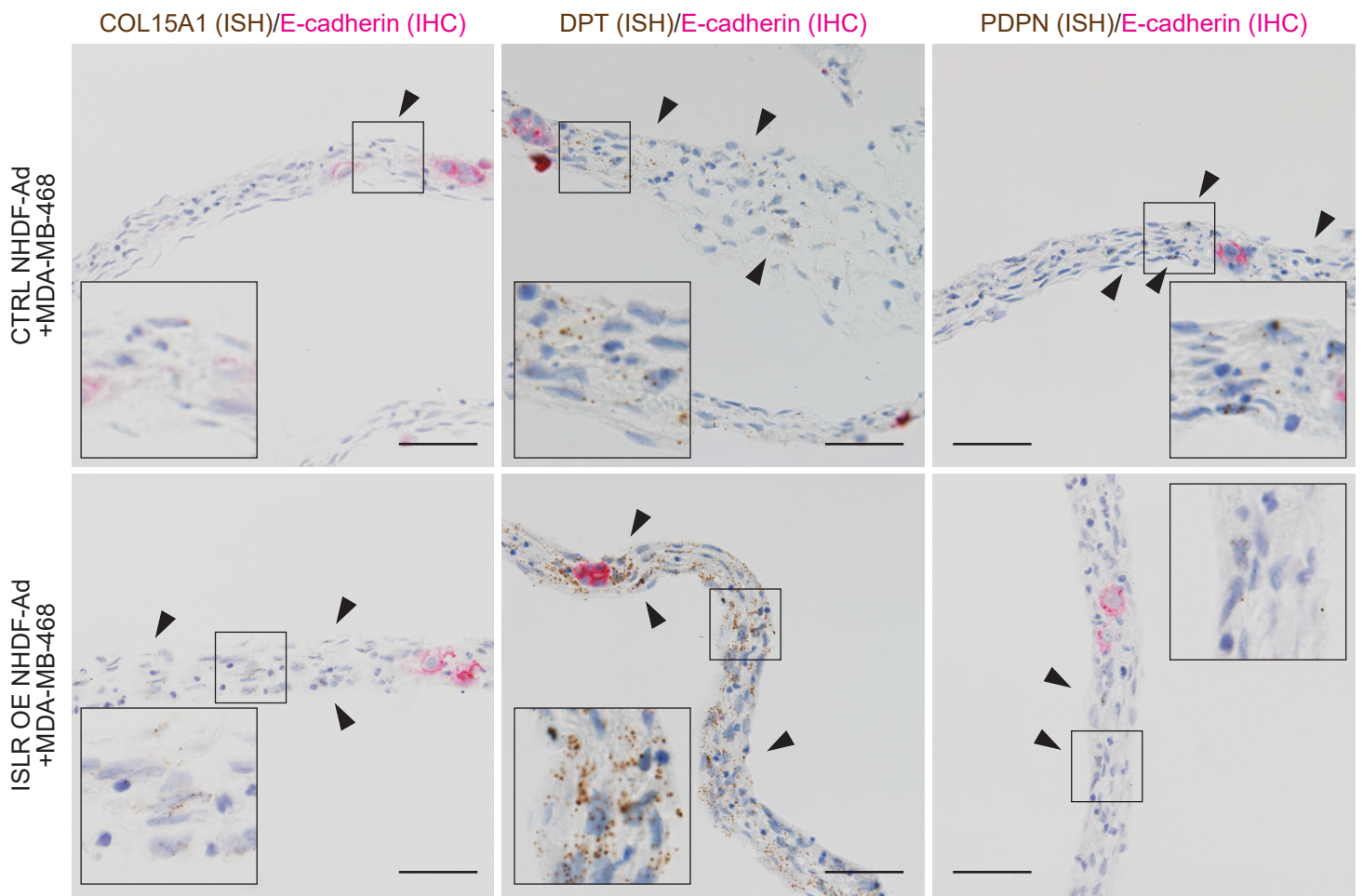

B

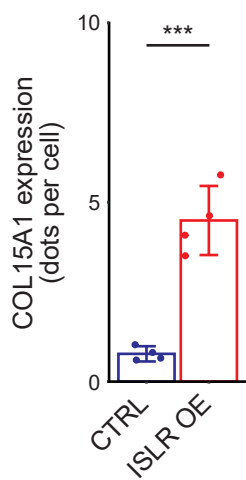

C

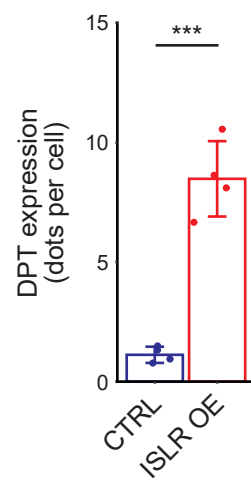

D

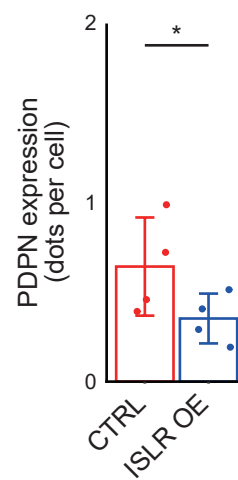

Supplement: Supplemental Figure S9 — Meflin overexpression (OE) induces changes in gene expressions of COL15A1, DPT, and podoplanin (PDPN) in fibroblasts co-cultured with MDA-MB-468 cells in a biomimetic three-dimensional (3D) culture system. A: Formalin-fixed, paraffin-embedded samples were prepared from 3D co-culture tumor models, followed by in situ hybridization (ISH) for COL15A1, DPT, or PDPN (brown) and immunohistochemistry (IHC) for E-cadherin (red). Arrowheads denote adult normal human dermal fibroblast (NHDF-Ad) cells positive for DPT, COL15A1, or PDPN. Boxed regions are shown at higher magnification (×2.5) in insets within the same images. B–D: The number of ISH signal dots for COL15A1 (B), DPT (C), or PDPN (D) per cell was counted and quantified. Data represent mean values of n = 4 samples per group. Error bars indicate standard deviation. ∗P < 0.05, ∗∗∗P < 0.001. Scale bars = 50 μm (A). ISLR, immunoglobulin superfamily containing leucine rich repeat. [file mmc9.pdf]

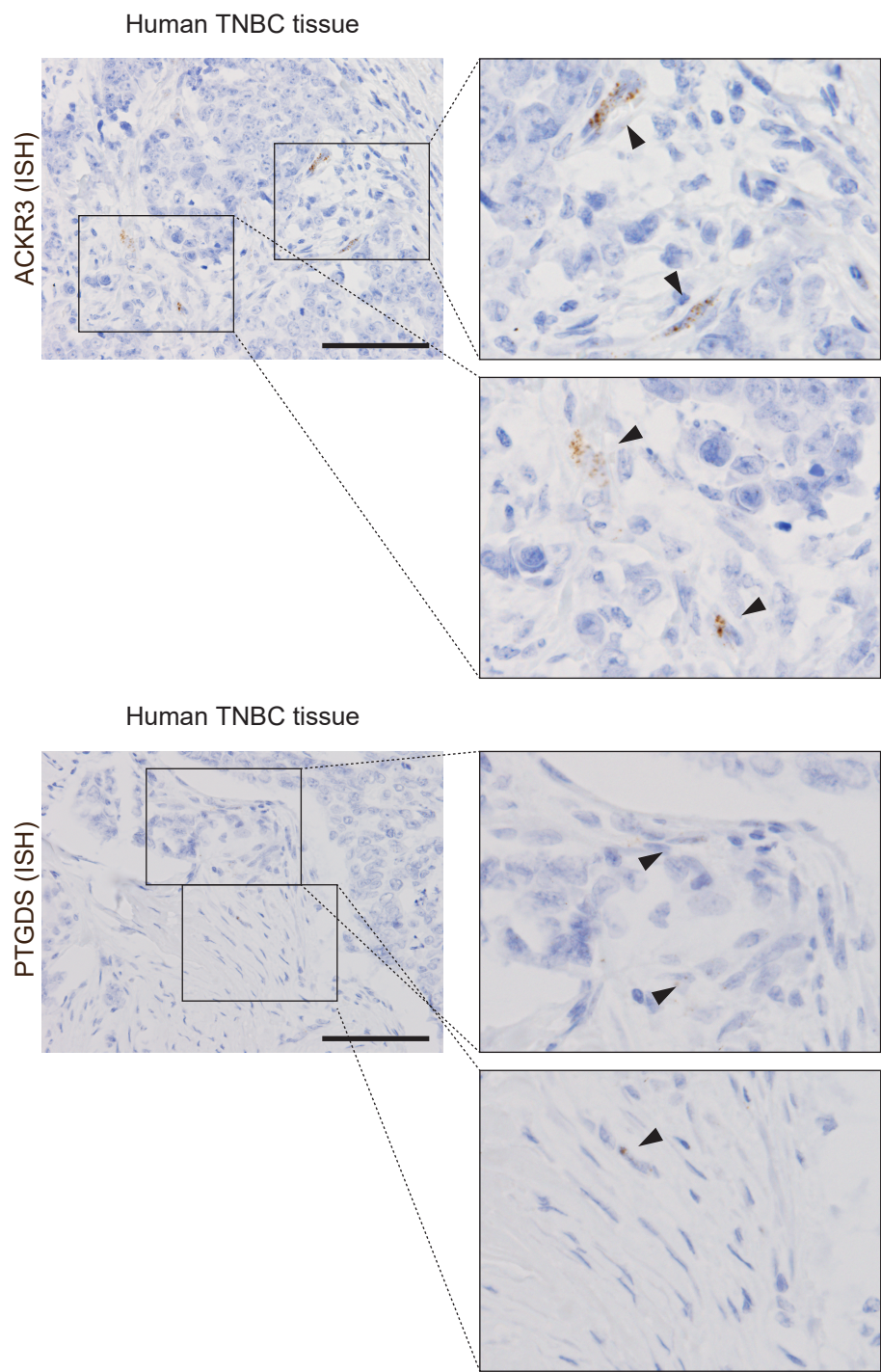

Supplement: Supplemental Figure S10 — ACKR3 and PTGDS are expressed in the stromal cells of human triple-negative breast cancer (TNBC). Tissue sections from human TNBC were stained for ACKR3 (upper panels) or PTGDS (lower panels) by in situ hybridization (ISH). Representative ISH images are presented. The boxed regions in the left panels are shown at higher magnification (×2.5) in the right panels. The arrowheads indicate ACKR3- or PTGDS-positive fibroblasts. Scale bars = 100 μm. [file mmc10.pdf]

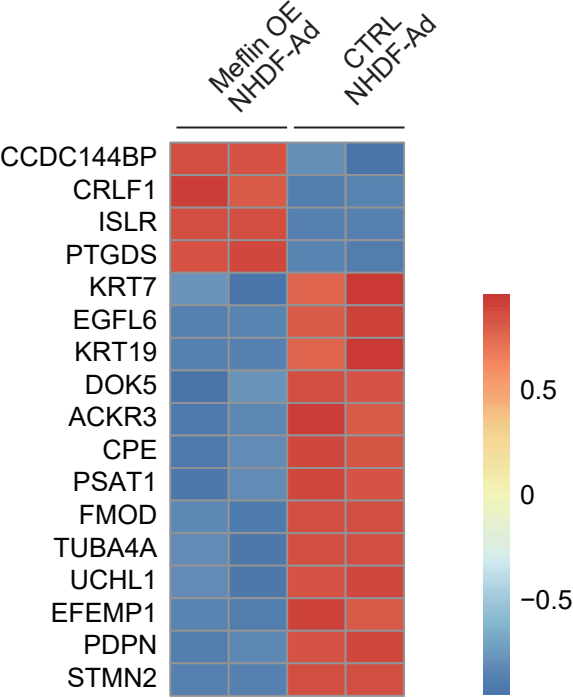

Supplement: Supplemental Figure S11 — RNA sequencing data from adult normal human dermal fibroblast (NHDF-Ad) cells cultured alone in a three-dimensional (3D) culture model using a cell-stacking technique. RNA sequencing was performed on total RNA samples isolated from Meflin overexpressing (OE) and control (CTRL) NHDF-Ad cells that were subjected to the 3D culture model. The heatmap displays 17 genes, consisting of the 15 highest variably expressed genes across samples and CCDC144BP and PTGDS, both of which were markedly altered also in co-culture with MDA-MB-468 cells. n = 2 per group. [file mmc11.pdf]

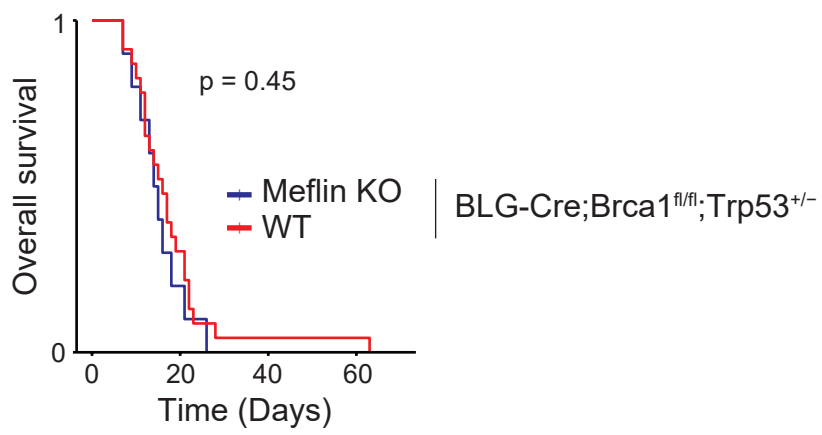

Supplement: Supplemental Figure S12 — Meflin deficiency did not affect overall survival of the triple-negative breast cancer mouse model. Survival rates of Meflin knockout (KO) and wild-type (WT) BLG-Cre;Brca1fl/fl;Trp53+/− mice were analyzed using the Kaplan-Meier method based on days after tumor formation. n = 10 KO mice; n = 23 WT mice. [file mmc12.pdf]
