# Supplementary material for: Increased Meflin Expression in Cancer-Associated Fibroblasts Restrains Tumor Cell Proliferation and Shapes Vessel-Rich Stroma in Triple-Negative Breast Cancer
Source: Am J Pathol. 2026 Feb 2;196(5):1205–19. doi: 10.1016/j.ajpath.2026.01.006 (PMC13197952; doi:10.1016/j.ajpath.2026.01.006)

Supplementary Figure S8

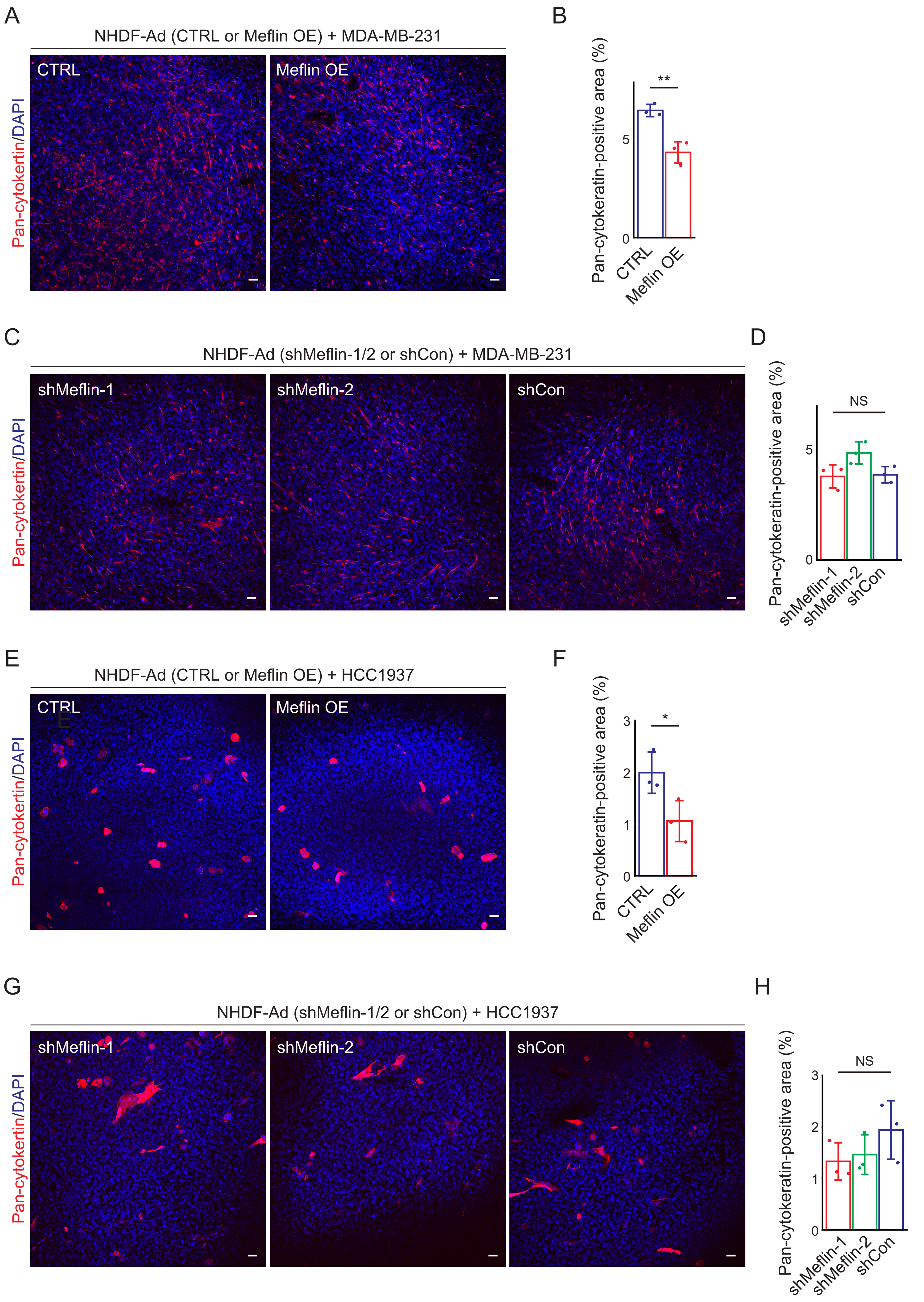

Supplement: Supplemental Figure S8 — Effects of Meflin overexpression (OE) and knockdown in fibroblasts on the proliferation of MDA-MB-231 and HCC1937 cells in a biomimetic three-dimensional (3D) culture system. A: Representative cross-sectional images of 3D co-culture of MDA-MB-231 cells and Meflin OE or control (CTRL) adult normal human dermal fibroblast (NHDF-Ad) cells. MDA-MB-231 cells were visualized using immunofluorescence staining for pan-cytokeratin (red). Nuclei were visualized using DAPI staining. B: Quantification of pan-cytokeratin–positive areas using ImageJ software version 1.8.0_172/1.53c in A. C: Representative cross-sectional images of 3D co-culture of MDA-MB-231 cells and Meflin knockdown (shMeflin-1 or -2) or CTRL (shControl [shCon]) NHDF-Ad cells. D: Quantification of pan-cytokeratin–positive areas using ImageJ software in C. E: Representative cross-sectional images of 3D co-culture of HCC1937 cells and Meflin OE or CTRL NHDF-Ad cells. F: Quantification of pan-cytokeratin–positive areas using ImageJ software in E. G: Representative cross-sectional images of 3D co-culture of HCC1937 cells and Meflin knockdown (shMeflin-1 or -2) or CTRL (shCon) NHDF-Ad cells. H: Quantification of pan-cytokeratin–positive areas using ImageJ software in G. Error bars indicate standard deviation. n = 3 per group (B, D, F, H). ∗P < 0.05, ∗∗P < 0.01. Scale bars = 50 μm (A, C, E, and G). NS, not significant. [file mmc8.pdf]
